# THOC3 interacts with YBX1 to promote lung squamous cell carcinoma progression through PFKFB4 mRNA modification

**DOI:** 10.1038/s41419-023-06008-3

**Published:** 2023-07-27

**Authors:** Tao Yu, Qi Zhang, Shao-Kun Yu, Feng-Qi Nie, Mei-Ling Zhang, Qian Wang, Kai-Hua Lu

**Affiliations:** 1 Department of Oncology, the First Affiliated Hospital of Nanjing Medical University, No. 300 Guangzhou Road, Nanjing, China; 2 Department of Oncology, the Affiliated Taizhou People’s Hospital of Nanjing Medical University, Taizhou, China; 3 Department of Oncology, the Second Affiliated Hospital of Nanjing Medical University, Nanjing, China

**Keywords:** Non-small-cell lung cancer, RNA

## Abstract

The THO complex (THOC) is ubiquitously involved in RNA modification and various THOC proteins have been reported to regulate tumor development. However, the role of THOC3 in lung cancer remains unknown. In this study, we identified that THOC3 was highly expressed in lung squamous cell carcinoma (LUSC) and negatively associated with prognosis. THOC3 knockdown inhibited LUSC cell growth, migration, and glycolysis. THOC3 expression was regulated by TRiC proteins, such as CCT8 and CCT6A, which supported protein folding. Furthermore, THOC3 could form a complex with YBX1 to promote PFKFB4 transcription. THOC3 was responsible for exporting PFKFB4 mRNA to the cytoplasm, while YBX1 ensured the stability of PFKFB4 mRNA by recognizing m5C sites in its 3′UTR. Downregulation of PFKFB4 suppressed the biological activities of LUSC. Collectively, these findings suggest that THOC3, folded by CCT proteins can collaborate with YBX1 to maintain PFKFB4 expression and facilitate LUSC development. Therefore, THOC3 could be considered as a novel promising therapeutic target for LUSC.

## Introduction

Lung cancer is a major public health concern with the highest morbidity and mortality rates globally. Non-small cell lung cancer (NSCLC) accounts for approximately 85% of all lung cancers, of which lung adenocarcinoma (LUAD) and lung squamous cell carcinoma (LUSC) are the two predominant subtypes. Targeted therapy and immunotherapy have emerged as promising treatment options for LUAD, but the same cannot be said for LUSC [1]. As LUSC accounts for almost 30% of all NSCLCs and lacks significant driver gene mutations, targeted therapy is not an efficient treatment [2]. Therefore, it is particularly important to investigate the molecular mechanism underlying LUSC progression to develop effective clinical regimens.

Recent studies have highlighted the pivotal role of the mRNA nuclear export in tumorigenesis and tumor progression [3]. mRNA export is a key step in gene expression and directly impacts the development of eukaryotic cells [4]. Transcription–Export (TREX) complex primarily mediates the transport of mature mRNAs from the nucleus to the cytoplasm. As a component of the TREX complex, the THO complex (THOC) is involved in the splicing, elongation, and nuclear export of nascent RNA. In mammals, THOC is comprised of six subunits: THOC1~3 and THOC5~7 [5]. THOC proteins cooperate with accessory proteins, including ALYREF (also known as THOC4), to facilitate the formation of mRNA ribonucleoparticle (mRNP) complexes and modulate the proliferation and differentiation status.

Aberrant expression of THOC alters the quality control of mRNA export and affects transcription. Knockdown of THOC induces transcriptional impairment and increases genomic instability [6]. Several THOC proteins have been reported to promote tumorigenesis in various malignancies. THOC1 is highly expressed in tumors and promotes the proliferation and aggressiveness of breast, lung, and prostate cancers [7, 8]. THOC2 and THOC5 enhance stemness and radio-resistance of triple-negative breast cancer [9]. However, the role of THOC3 in tumor has not been systematically studied. THOC3 appears to play a prominent role during the early stages of differentiation [10]. A wide spectrum of transcription factors is regulated by THOC3 in fish. It has been reported that THOC3 highly expressed in glioma cells predicts poor prognosis [11]. Here, we identified the high expression of THOC3 in LUSC, and demonstrated that THOC3 knockdown inhibited LUSC cell growth. We also detected the binding between THOC3 and YBX1, and showed that they can cooperate to induce the expression of PFKFB4. These results suggest that the THOC3/YBX1/PFKFB4 axis promotes LUSC development.

## Results

### THOC3 is highly expressed in LUSC

**Fig. 1:**
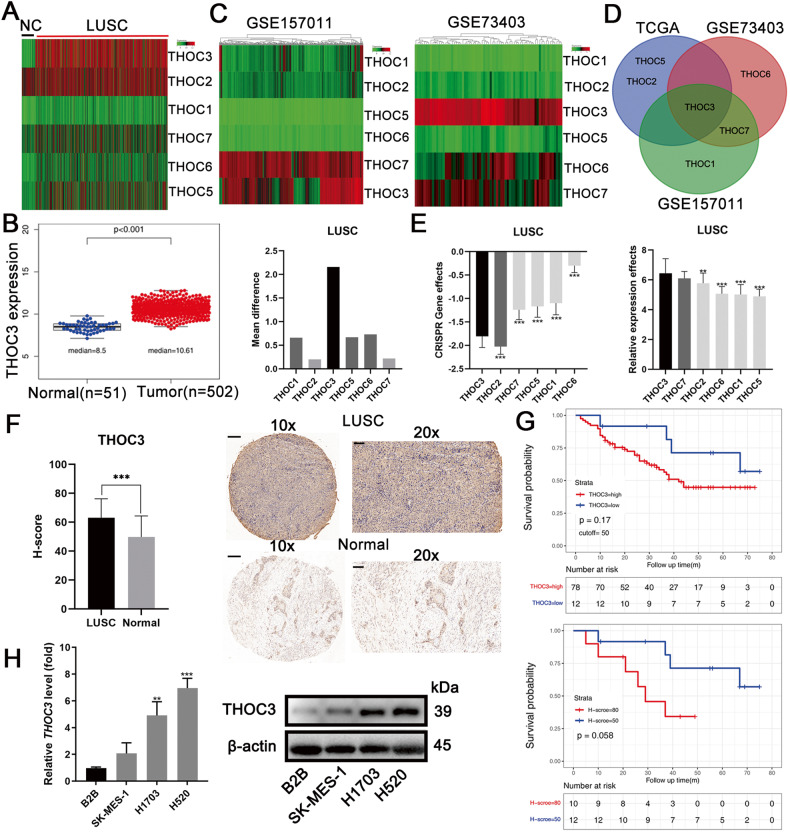
THOC3 is highly expressed in LUSC. **A** Heatmap of THOC gene expression levels in LUSC (*n* = 502) and normal tissues (*n* = 51) based on TCGA data. **B** Bar chart showing the difference in mean THOC expression levels between LUSC and normal tissues (right) and the specific expression levels of THOC3 in LUSC and normal tissues (left). **C** Heatmaps of THOC levels in LUSC datasets GSE157011 (*n* = 484) and GSE73403 (*n* = 69). **D** Venn diagram showing the three most highly expressed genes in the THOC family according to TCGA, GSE157011, and GSE73403. **E** DEPMAP analysis of the effects of THOC1/2/3/5/6/7 knockout on 22 LUSC cell lines (left) with THOC levels in 27 LUSC cell lines shown on the right. **F** Immunohistochemistry (IHC) staining of THOC3 in 90 pairs of LUSC tissues and adjacent normal tissues. Scale bars = 100 μm. **G** Kaplan-Meier plots of patients with high (H-score > 50) or low (H-score ≤ 50) THOC3 expression based on TMA results (top), and patients with H-score ≥ 80 or ≤50 based on TMA results (bottom). **H** THOC3 mRNA and protein expression levels in B2B, SK-MES-1, H1703, and H520 shown by qRT-PCR and WB. *P < 0.05; **P < 0.01; ***P < 0.001. Variables are presented as mean ± SD.

Using data from The Cancer Genome Atlas (TCGA), differential expression of THOC genes was identified between normal lung and LUSC tissues (Fig. 1A). Among all THOC family members, THOC3 showed the most significant difference in expression (Fig. 1B, Supplementary Fig. S1A). Additionally, analysis of GSE157011 and GSE73403 showed that THOC3 was highly expressed in LUSC (Fig. 1C). Patients with high expression of THOC2, THOC3, or THOC5 had a poorer prognosis than those with low expression (Supplementary Fig. S1B). After analyzing the three most highly expressed THOC genes in TCGA, THOC3 was selected for further investigation (Fig. 1D). CRISPR/Cas9 screening of 22 LUSC cell lines obtained from the DEPMAP database revealed the importance of the THOC family, with THOC3 knockout causing significant cell growth defects, and THOC3 expression was significantly higher than that of THOC1/2/5/6 in 27 LUSC cell lines (Fig. 1E). Pan-cancer analysis showed that THOC3 is pervasively overexpressed in multiple malignancies compared with normal tissues (Supplementary Fig. S1C). In total, 90 pairs of LUSC tumors and adjacent normal tissues were collected and the clinical information of samples is presented in Table 1. THOC3 expression was higher in LUSC tissues than in normal tissues shown via immunohistochemical (IHC), and patients with high H-scores had relatively worse prognoses (Fig. 1F, G). Patients with the lowest 25% of the H-score and those with the highest 25% of the H-score showed considerably significant differences in survival. Quantitative reverse transcription polymerase chain reaction (qRT-PCR) and Western blotting (WB) analysis were performed to confirm the overexpression of THOC3 in LUSC cell lines (H1703, H520, and SK-MES-1) compared with the pulmonary epithelial cell B2B (Fig. 1H).

**Table 1:** Clinical information of 90 patients with LUSC.

Parameter	Total [cases (%)]	THOC3 Expression [cases (%)]		*P* value
		Low^a^	High^a^	
Total	90	12 (13.3%)	78 (86.7%)	
Gender				
Male	87 (96.7%)	12 (100.0%)	75 (96.2%)	0.490
Female	3 (3.3%)	0 (0.0%)	3 (3.3%)	
Age at surgery (years)				
≥65	57 (63.3%)	8 (66.7%)	49 (62.8%)	0.797
<65	33 (36.7%)	4 (33.3%)	29 (37.2%)	
Grade				
High	5 (5.6%)	1 (8.3%)	4 (5.1%)	0.520
Middle and Low	85 (94.4%)	11 (91.7%)	74 (94.9%)	
Pathological stage				
I	25 (27.8%)	3 (25.0%)	22 (28.2%)	0.817
II–IV	65 (72.2%)	9 (75.0%)	56 (71.8%)	
Tumor size (cm)				
<4	38 (42.2%)	3 (25.0%)	35 (44.9%)	0.194
≥4	52 (57.8%)	9 (75.0%)	43(55.1%)	
Lymph node metastasis				
Absent	49 (54.4%)	6 (50.0%)	43 (55.1%)	0.765
Present	41 (45.6%)	6 (50.0%)	35 (44.9%)	

^a^Low: H-score ≤ 50; High: H-score > 50.

### THOC3 promotes the proliferation, migration, and glycolysis of LUSC

**Fig. 2:**
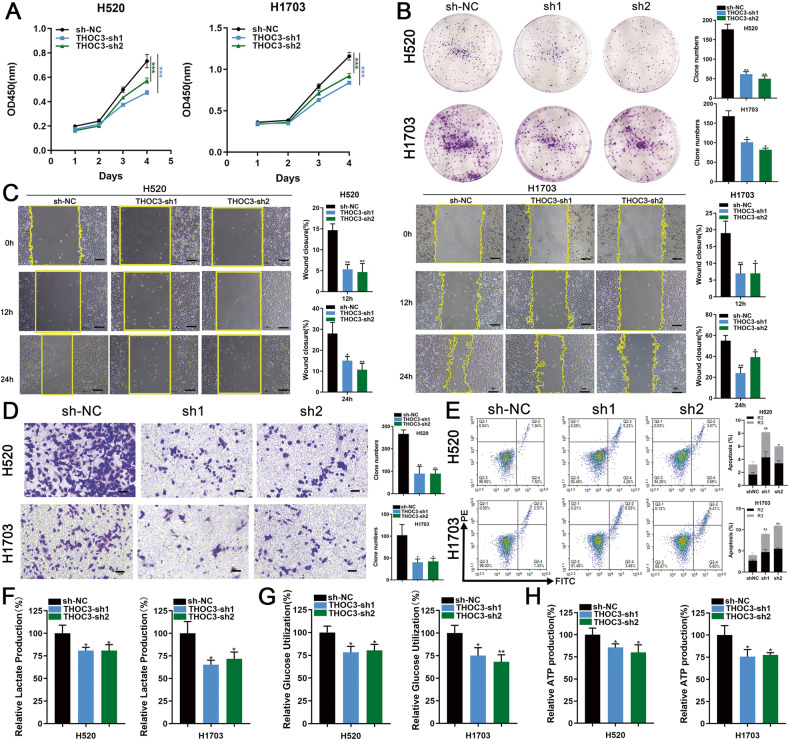
THOC3 promotes the proliferation, migration, and glycolysis of LUSC. **A**, **B** Growth curves (days 1–4) and colony formation assays represent the proliferation of H520/H1703 cells infected with shNC or THOC3-sh1/2. Representative images of the crystal violet staining of cells in a six-well plate are shown. **C** Migration of H520/H1703 cells infected with shNC or THOC3-sh1/2 was assessed using wound healing assays. Scratches were observed at 12 h and 24 h (10× magnification). **D** Transwell assays were performed to assess the migration of H520/H1703 cells with THOC3 knockdown. Cells crossing the membrane were dyed with crystal violet (10× magnification). Scale bars = 100 μm. **E** THOC3 knockdown resulted in increased apoptosis in LUSC cells. Representative FACS images and statistics based on three independent experiments are shown. **F–H** The impact of THOC3 knockdown on glucose uptake, lactic acid and ATP production in H520/H1703 cells after 24 h was evaluated. *P < 0.05; **P < 0.01; ***P < 0.001. Variables are presented as mean ± SD.

H1703 and H520 cell lines exhibited reduced THOC3 expression upon the administration of short hairpin RNAs (shRNAs) (Supplementary Fig. S2A). THOC3 knockdown suppressed the OD values and colony formation of LUSC cell lines (Fig. 2A, B). Besides, wound healing and transwell assays showed that THOC3 knockdown decreased cell migration (Fig. 2C, D). Furthermore, the fraction of apoptotic cells significantly increased following THOC3 knockdown (Fig. 2E). RNA sequencing (RNA-seq) was performed to evaluate the impact of THOC3 knockdown on H1703. Gene Set Enrichment Analysis (GSEA) revealed that the glycolysis pathway was suppressed after THOC3 knockdown (Supplementary Fig. S2B). Subsequent investigation into the effect of THOC3 on glucose metabolism demonstrated that THOC3 knockdown reduced glucose utilization, lactic acid production, and intracellular ATP level (Fig. 2F–H) in H520 and H1703 cells. These results indicated that THOC3 promoted LUSC progression.

### THOC3 expression affects LUSC growth in vivo

**Fig. 3:**
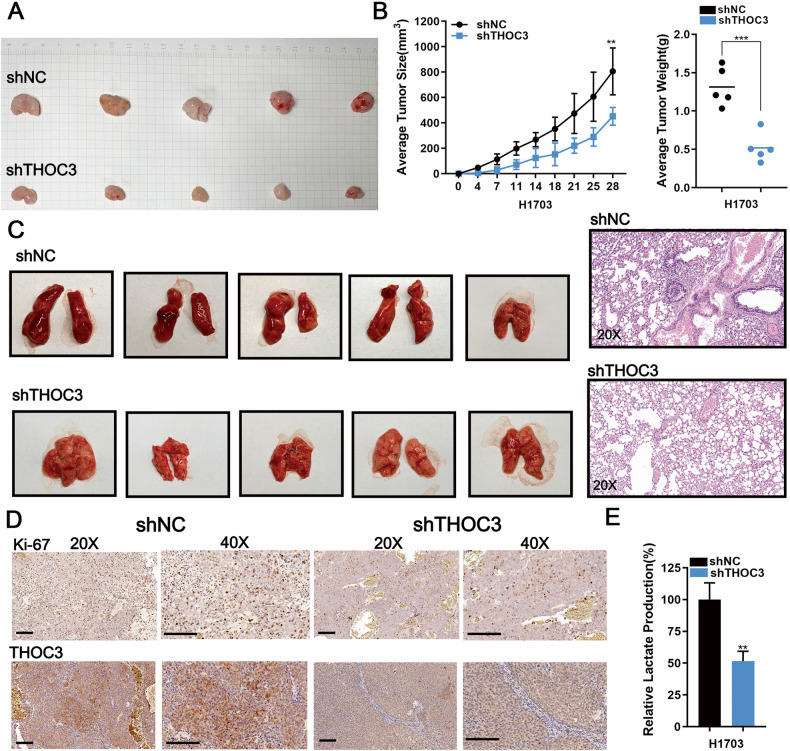
Loss of THOC3 inhibits LUSC growth in vivo. **A** Representative images of subcutaneous tumors on the left flank of mice injected with 1 × 10^6^ shNC/shTHOC3 H1703 cells (*n* = 5, each group). **B** The mean weight of each tumor is measured (left); tumor growth curves are plotted every 2-3 days. **C** Representative images of lungs from shNC/shTHOC3 groups after tail vein injection (1 × 10^6^ cells/100 μl) for 1 month (*n* = 5, each group) (top). Hematoxylin-eosin staining images of different lungs (20× magnification) (bottom). **D** IHC shows the levels of Ki-67 index and THOC3 in shNC/shTHOC3 groups (20× and 40× magnification). Scale bars = 100 μm. **E** Lactic acid production of the THOC3 knockdown group compared to the shNC group. *P < 0.05; **P < 0.01; ***P < 0.001. Variables are presented as mean ± SD.

We investigated the impact of THOC3 in vivo on tumorigenicity of LUSC. LUSC cells were injected into BALB/c-nude mice, and THOC3 knockdown was found to inhibit the increase in tumor volume and weight, as well as suppress lung metastasis (Fig. 3A–C). THOC3 silencing also decreased the Ki-67 index (Fig. 3D). A considerable reduction in the lactic acid production was also observed in the THOC3 knockdown group (Fig. 3E). Conversely, tumors from THOC3-overexpressed LUSC cell lines grew faster than those from the control group, with higher Ki-67 index, and increased lactic acid production (Supplementary Fig. S3A–D). Thus, THOC3 plays a tumor-promoting role in vivo.

### THOC3 is located in cytoplasm and folded by CCT

**Fig. 4:**
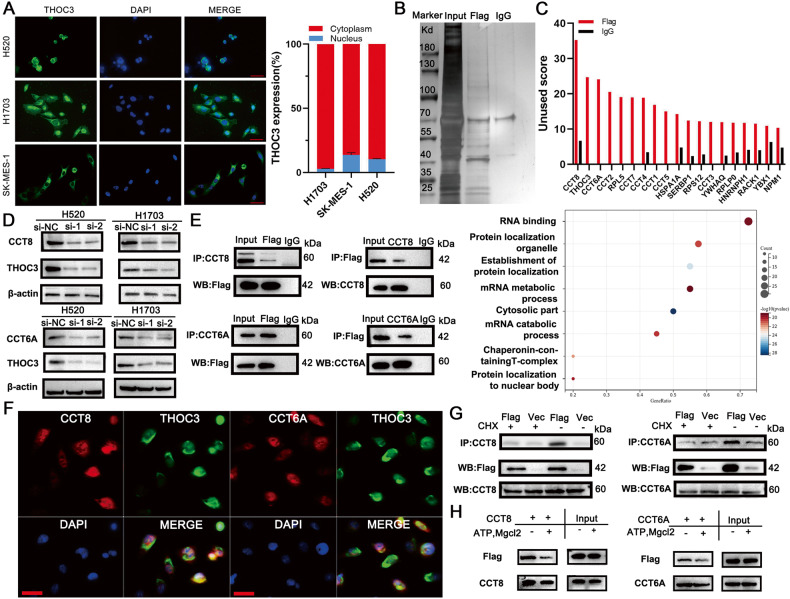
THOC3 is located in cytoplasm and folded by CCT. **A** Representative images of THOC3 localization in H520/H1703/SK-MES-1 cells detected by Fluorescent In Situ Hybridization (FISH) (40× magnification), scale bars = 100 μm (left); THOC3 mRNA expression in the nucleus and cytoplasm was determined via qRT-PCR in H1703/SK-MES-1/H520 cells (right). **B** Silver staining of immunoprecipitation of Flag and IgG antibodies in H1703 cells. **C** Bar chart showing proteins with unused scores of Flag over 10, identified via MS analysis of H1703 cells following immunoprecipitation with Flag and IgG antibodies (top); representative signaling pathways composed of proteins bound to THOC3 based on GO analysis (bottom). **D** THOC3 expression in cells transfected with si-NC or si-CCT8/CCT6 shown by WB. **E** Immunoprecipitation of cell lysates from H1703 cells transfected with Flag-tagged THOC3 using anti-CCT8, anti-CCT6A, anti-Flag, or IgG antibody, and immunoprecipitates were blotted with anti-Flag/CCT8/CCT6A antibodies. **F** Immunofluorescence images showing the distribution of THOC3 (green) and CCT8/CCT6A (red) in H1703 cells. Scale bars = 100 μm. **G** Cells are transfected with Flag-tagged THOC3 for 18 h and then incubated with and without 50 μg/ml cycloheximide (CHX) for 5 h. Immunoprecipitates were blotted with anti-CCT8/CCT6A antibodies. **H** Cells expressing Flag tagged THOC3 are prepared in the presence or absence of ATP and MgCl_2_ and analyzed via immunoprecipitation and WB by anti-Flag/CCT8/CCT6A antibodies.

Immunofluorescence (IF) staining and nuclear/cytoplasmic separation experiments confirmed the cytoplasmic localization of THOC3 (Fig. 4A). To elucidate the mechanism underlying the functions of THOC3, mass spectrometry (MS) analysis was performed to identify proteins that interacted with THOC3 (Fig. 4B, Supplementary Table S2). Gene ontology functional enrichment analysis revealed that THOC3-binding proteins were involved in RNA binding, mRNA metabolic process, and mRNA catabolic process, which are consistent with functions of the THOC family (Fig. 4C).

Analysis of MS data revealed that TRiC (tailless complex protein-1 [TCP-1] ring complex), composed of CCT1-8, interacted with THOC3 (Fig. 4C). CCT8, CCT6A, and CCT2 were found to be the top three proteins associated with THOC3. CCT8 and CCT6A were essential for TRiC function and hypothesized to regulate the folding of THOC3. THOC3 expression correlated with CCT8/6A/2/5 in TCGA (Supplementary Fig. S4A). Knockdown of CCT8 or CCT6A decreased the expression of THOC3 protein (Fig. 4D), while their knockdown had no effects on THOC3 mRNA (Supplementary Fig. S4B). Co-immunoprecipitation (co-IP) showed that CCT8, CCT6A, and CCT2 could bind to Flag-tagged THOC3 (Fig. 4E, Supplementary Fig. S4C). Confocal fluorescent microscopy indicated the co-localization of THOC3 and CCT8 or CCT6A (Fig. 4F). Treatment with cycloheximide for 5 h abolished the binding of CCT8 and CCT6A to THOC3, suggesting that CCT proteins preferentially interacted with newly synthesized THOC3 (Fig. 4G). In the presence of ATP and Mg^2+^, the expression of the Flag-THOC3 that coprecipitated with CCT8/CCT6A was reduced (Fig. 4H). These results suggested that THOC3 was a TRiC client protein.

### THOC3 promotes LUSC partially through PFKFB4 by binding to YBX1

**Fig. 5:**
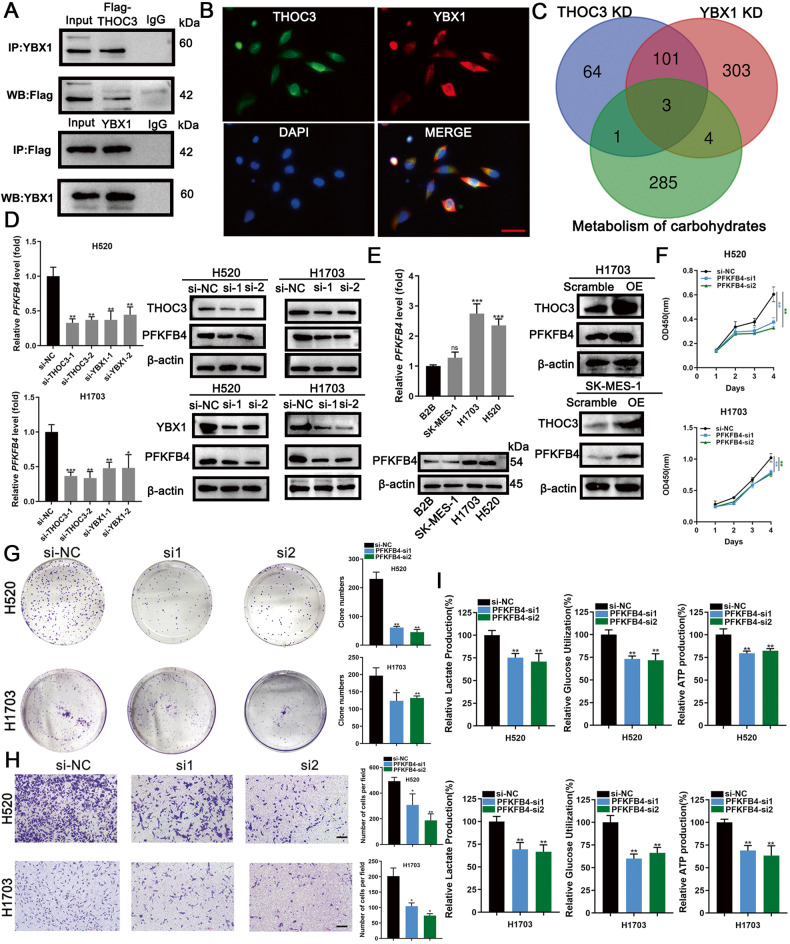
THOC3 promotes LUSC partially through PFKFB4 by binding to YBX1. **A** Cell lysates from H1703 cells transfected with Flag-tagged THOC3 were immunoprecipitated with anti-YBX1, anti-Flag, or IgG antibodies, and the immunoprecipitates were blotted with anti-Flag/YBX1 antibodies. **B** Immunofluorescence shows the distribution of THOC3 (green) and YBX1 (red) in H1703. Scale bars = 100 μm. **C** Genes significantly downregulated upon THOC3 or YBX1 knockdown (KD) and associated with the carbohydrate metabolism (Venn diagram). **D** Outcomes of qRT-PCR and WB showing PFKFB4 expression in LUSC cells transfected with si-NC or si-THOC3/YBX1. **E** PFKFB4 levels in B2B, SK-MES-1, H1703, and H520 are shown using qRT-PCR and WB (left); PFKFB4 expression in cells transfected with scramble or THOC3-OE plasmids shown by WB (right). **F**, **G** Growth curves (day 1–4) and colony formation assays show the proliferation of H520/H1703 cells transfected with si-NC or PFKFB4-si1/2. **H** Transwell assays were performed to assess the migration of H520/H1703 cells with PFKFB4 knockdown. Cells crossing the membrane were dyed with crystal violet (10× magnification). Scale bars = 100 μm. **I** The relative glucose uptake, lactic acid and ATP production of H520/H1703 cells transfected with si-NC or PFKFB4-si1/2 after 24 h. *P < 0.05; **P < 0.01; ***P < 0.001. Variables are presented as mean ± SD.

MS analysis also revealed that THOC3 bound to YBX1, an important protein involved in mRNA stabilization. The combination between THOC3 and YBX1 was further confirmed via co-IP and IF experiments (Fig. 5A, B). WB results showed that the level of YBX1 remained unchanged in THOC3 knockdown LUSC cell lines, revealing that YBX1 is a binding partner of THOC3, rather than a downstream molecule (Supplementary Fig. S5A). To identify common downstream targets of THOC3 and YBX1, RNA-seq was performed, and 104 genes were significantly downregulated upon knockdown of either THOC3 or YBX1 (Supplementary Table S3). Considering the known effect of THOC3 on glycolysis, 104 genes were further overlapped with a gene profile of carbohydrate metabolism and several genes were screened out (PFKFB4, ENO2, and SLC2A1) (Fig. 5C). RNA-seq results showed that PFKFB4, ENO2, and SLC2A1 were significantly downregulated upon THOC3 or YBX1 knockdown, whereas other glycolysis-related genes, such as NRF2 and FOXM1, were not affected [12, 13] (Supplementary Fig. S5B). More PFKFB4 mRNA was reduced than ENO2 or SLC2A1 mRNA shown by qRT-PCR (Fig. 5D, Supplementary Fig. S5C). The downregulation of PFKFB4 was also confirmed by WB (Fig. 5D). In subcutaneous tumor models, THOC3 knockdown inhibited the expression of PFKFB4, and THOC3 overexpression increased its level (Supplementary Fig. S5D).

According to TCGA, PFKFB4 was overexpressed in LUSC (Supplementary Fig. S5E). Bioinformatics analysis indicated that PFKFB4 expression was positively proportional to THOC3 or YBX1 expression (Supplementary Fig. S5F). Additionally, CRISPR/Cas9 screening in DEPMAP confirmed the necessity of PFKFB4 in LUSC cell lines (Supplementary Fig. S5G). LUSC cell lines (H1703 and H520) expressed more PFKFB4 than B2B, and THOC3 overexpression upregulated the level of PFKFB4 (Fig. 5E). PFKFB4 expression was decreased by small interfering RNA (siRNA) (Supplementary Fig. S6A). Knockdown of PFKFB4 inhibited proliferation, colony formation (Fig. 5F, G), and migration of LUSC cells (Fig. 5H), and also reduced glucose utilization, lactic acid production, and intracellular ATP level (Fig. 5I), consistent with its role in enhancing glucose metabolism and promoting LUSC development.

### Oncogenic role of THOC3 depends on PFKFB4

**Fig. 6:**
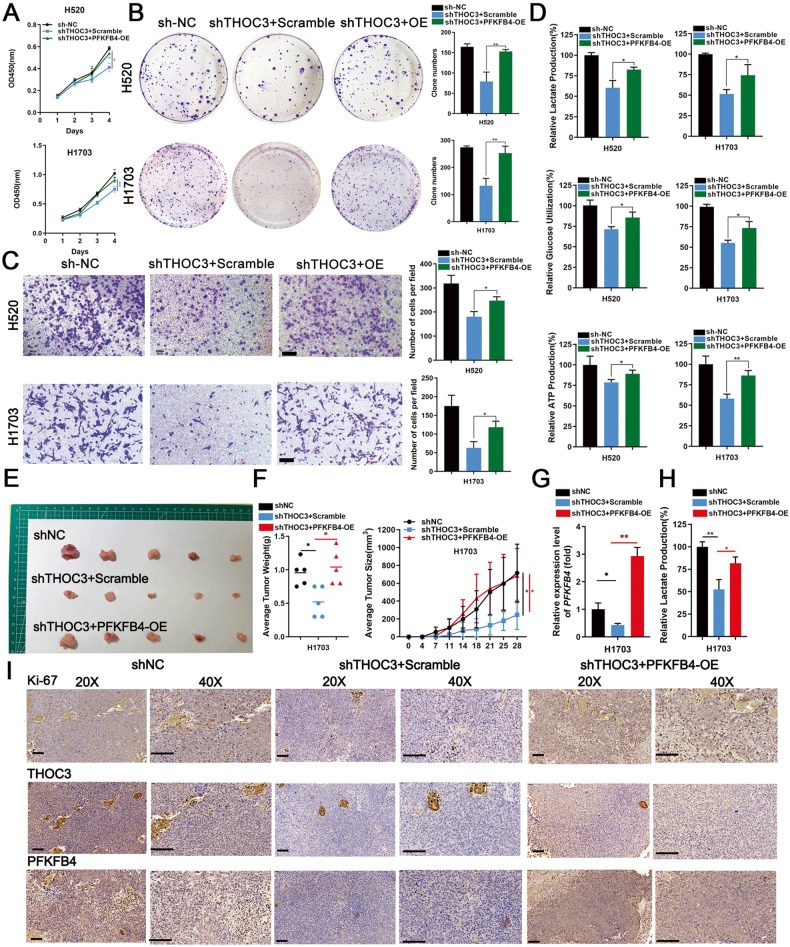
Oncogenic role of THOC3 depends on PFKFB4. **A**, **B** Growth curves (day 1–4) and colony formation assays depict the proliferation of H520/H1703 cells treated with shNC or shTHOC3 plus scramble/PFKFB4-OE plasmids. **C** Transwell assays show migration of shNC/shTHOC3 infected H520/H1703 cells with scramble/PFKFB4-OE (10× magnification). Scale bars = 100 μm. **D** The glucose uptake, lactic acid and ATP production of shNC/shTHOC3 infected H520/H1703 cells with scramble/PFKFB4-OE after 24 h. **E**–**F** Representative images of subcutaneous tumors on the left flank of mice injected with 1 × 10^6^ shNC/shTHOC3 infected H1703 cells with scramble or PFKFB4-OE plasmids (*n* = 5, each group). The mean weight of each tumor is measured, and tumor growth curves are plotted every 2–3 days. **G** PFKFB4 mRNA in shNC/shTHOC3 infected H1703 cells with scramble or PFKFB4-OE plasmids was shown by qRT-PCR. **H** Lactic acid production of the shTHOC3 groups transfected with scramble or PFKFB4-OE plasmids compared to the shNC group. **I** IHC shows the levels of Ki-67 index, THOC3, and PFKFB4 in three groups (20× and 40× magnification). Scale bars = 100 μm. *P < 0.05; **P < 0.01; ***P < 0.001. Variables are presented as mean ± SD.

To further investigate the relationship between THOC3 and PFKFB4, PFKFB4 overexpression or scramble vectors were transfected into THOC3-knockdown H520 and H1703 cell lines. The protein expression of PFKFB4 in transfected cells was confirmed by WB (Supplementary Fig. S6B). CCK-8 and colony formation assays demonstrated that PFKFB4 overexpression rescued cell proliferation (Fig. 6A, B). In addition, upregulated PFKFB4 promoted cell migration (Fig. 6C). The ectopic expression of PFKFB4 restored downregulated glucose utilization, lactic acid production, and intracellular ATP levels caused by THOC3 knockdown (Fig. 6D). In mouse subcutaneous tumor models, knockdown of THOC3 inhibited the proliferation of tumor. However, PFKFB4 overexpression could rescue the reduction of tumor growth (Fig. 6E–G). Similarly, PFKFB4 upregulation restored the downregulated lactate production and Ki-67 caused by THOC3 downregulation (Fig. 6H, I). Additionally, treatment with the PFKFB4 inhibitor 5MPN decreased the expression of PFKFB4 in THOC3-overexpressed LUSC cell lines (Supplementary Fig. S6C). Rescue assays showed that THOC3 overexpression promoted the growth, migration, and glycolysis, while the utilization of 5MPN impaired the biological activities of LUSC cells (Supplementary Fig. S6D–F). Results of rescue experiments indicated that PFKFB4, as the downstream of THOC3, could mediate tumor-promoting effects in LUSC cells.

### PFKFB4 mRNA is exported by THOC3 and stabilized by YBX1

**Fig. 7:**
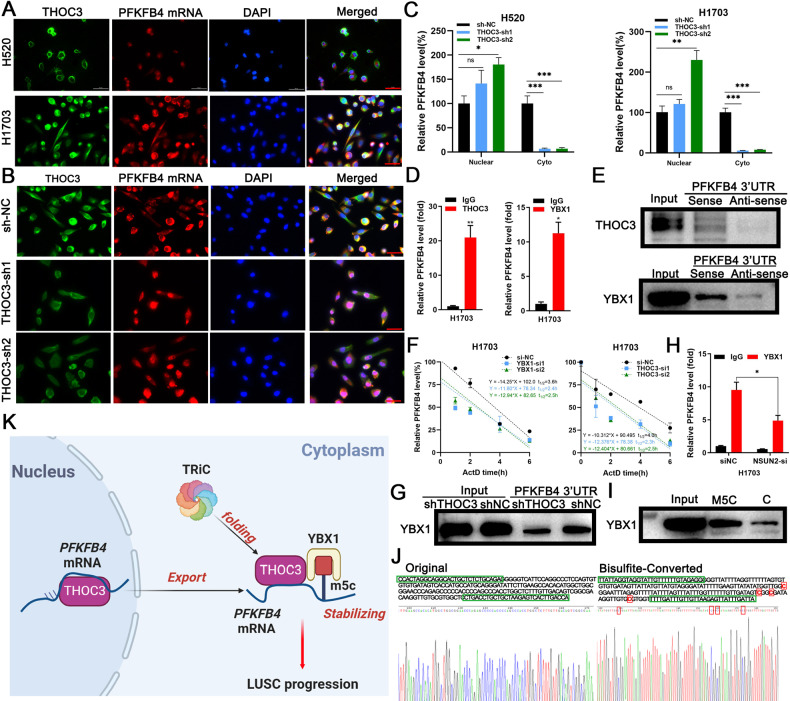
PFKFB4 mRNA is exported by THOC3 and stabilized by YBX1. **A** FISH images show THOC3 and PFKFB4 mRNA localization in H520/H1703 cells (40× magnification). Scale bars = 100 μm. **B** FISH images show localization of THOC3 and PFKFB4 mRNA in shNC/shTHOC3 H1703 cells (40× magnification). Scale bars = 100 μm. **C** qRT-PCR analysis shows PFKFB4 mRNA expression in the nucleus and cytoplasm of THOC3 knockdown H520/H1703 cells compared to shNC-infected cells. **D** Enrichment of PFKFB4 mRNA in THOC3/YBX1 immunoprecipitates relative to IgG in H1703 shown using qRT-PCR. **E** RNA pull-down analysis followed by WB shows the binding between the biotin-labeled sense strand of PFKFB4 3′UTR and THOC3/YBX1 in H1703 cells, with no binding detected with the anti-sense strand. **F** Detection of PFKFB4 mRNA at indicated time points after actinomycin D treatment in H1703 cells transfected with si-NC, YBX1-si1/2, or THOC3-si1/2. **G** RNA pull-down analysis followed by WB shows the binding between the biotin-labeled PFKFB4 3′UTR and YBX1 in shNC/shTHOC3 infected H1703 cells. **H** qRT-PCR results show PFKFB4 mRNA enriched in YBX1 immunoprecipitates relative to the IgG in H1703 transfected by si-NC or si-NSUN2. **I** Biotin-labeled RNA pull-down and WB in H1703 cells show the binding between YBX1 and regions of PFKFB4 3′UTR with or without m5C modification. **J** Sanger sequencing results show parts of normal PFKFB4 sequences (left) and sequences after bisulfite conversion (right), where methylated C is framed in red. **K** TRiC folds THOC3 protein, which binds to PFKFB4 mRNA and brings it out of the nucleus. Upon arriving in the cytoplasm, THOC3 acts as a scaffold to recruit YBX1 to stabilize the transcript by recognizing m5C. THOC3 and YBX1 work together to promote PFKFB4 mRNA transcription. Overexpressed PFKFB4 increases glycolysis and promotes LUSC proliferation and migration. *P < 0.05; **P < 0.01; ***P < 0.001. Variables are presented as mean ± SD.

Given the role of THOC in mRNA export, we investigated whether THOC3 was responsible for exporting PFKFB4 mRNA to the cytoplasm. Immunofluorescence revealed the colocalization of THOC3 and PFKFB4 mRNA in the cytoplasm (Fig. 7A, Supplementary Fig. S7A). Subsequent experiments showed that THOC3 knockdown caused the accumulation of PFKFB4 mRNA in the nucleus (Fig. 7B), and significantly decreased its expression in the cytoplasm but not in the nucleus (Fig. 7C). RNA immunoprecipitation (RIP) was performed to identify the binding between THOC3 and PFKFB4 mRNA (Fig. 7D). The results showed that PFKFB4 mRNA was significantly bound by THOC3 in comparison to IgG. The catRAPID tool predicted that THOC3 binding sites were enriched in 3′UTR of PFKFB4 mRNA (Supplementary Fig. S7B). RNA pull-down assays further confirmed the binding of THOC3 to the sense strand of the 3′UTR (Fig. 7E).

Once brought to the cytoplasm by THOC3, PFKFB4 mRNA was proposed to be stabilized by YBX1. Previous studies have identified potential binding sites of YBX1 to PFKFB4 mRNA through RIP-seq [14, 15]. To confirm this association, RIP and RNA pull-down assays were conducted in H1703 cells, revealing the binding of YBX1 to PFKFB4 mRNA (Fig. 7D, E). Knockdown of YBX1 or THOC3 significantly reduced the half-life of PFKFB4 mRNA in LUSC cell lines treated with actinomycin D (Fig. 7F). However, upon YBX1 knockdown, neither THOC3 knockdown nor overexpression rescued the half-life of PFKFB4 mRNA (Supplementary Fig. S7C), indicating that THOC3 indirectly stabilized PFKFB4 transcription through YBX1, which is a critical m5C reader and responsible for maintaining mRNA stability. RNA pull-down assays showed that the binding between YBX1 and PFKFB4 mRNA was weakened upon THOC3 knockdown (Fig. 7G). Besides, the expressions of PFKFB4 and NSUN2, the m5C writer, showed a positive correlation (Supplementary Fig. S5F), suggesting that the stabilization of PFKFB4 mRNA depends on m5C modification. RIP analysis revealed that NSUN2 siRNA transfection considerably decreased the binding efficiency of YBX1 to PFKFB4 mRNA in H1703 cells (Fig. 7H). RNA pull-down assays showed that probes of the binding sites with m5C modification had a higher binding affinity to YBX1 than those without m5C modification (Fig. 7I). Bisulfite treatment was utilized to detect m5C sites on PFKFB4 mRNA, revealing the presence of m5C modification primarily in CpG regions of the 3′UTR (Fig. 7J, Supplementary Fig. S7E). These results support that after PFKFB4 mRNA is delivered to the cytoplasm, THOC3 could recruit YBX1, which recognizes the m5C modification, to further maintain transcript stabilization (Fig. 7K).

## Discussion

Nowadays, it is well-established that the export of proteins, primarily mediated by the karyopherin XPO1, has a profound effect on tumor progression [16]. The XPO1 inhibitor KPT-330 has been approved for the treatment of multiple myeloma [17]. However, the role of mRNA export in tumor development requires further investigation. In this study, we aimed to identify the role of transcription export proteins in regulating LUSC progression. We found that THOC3 as a member of THO complex is involved in LUSC pathogenesis and characterized its function. THOC3 was highly expressed in both LUSC cell lines and clinical tissues, and its high expression predicted poor survival in early stage LUSC patients. Knockdown of THOC3 suppressed the proliferation and migration of LUSC cells, and induced apoptosis. GSEA results suggested that THOC3 promoted the glycolysis, which was confirmed by the significant inhibition of glucose utilization, lactic acid production, and intracellular ATP production upon THOC3 knockdown. Furthermore, we demonstrated the tumor-promoting role of THOC3 in vivo. Given that THOC3 is upregulated in LUSC and predicts poor prognosis, identifying its inhibitor might be of great significance for new drug development.

Our mass spectrometry results revealed that CCT proteins 1–8 could bind to THOC3, and their expression and the THOC3 level were positively correlated in TCGA. CCT 1–8 are eight homologous but distinct subunits that could form a large complex called TRiC, which has two interior chambers to encapsulate and fold substrate proteins. TRiC is a eukaryotic primary cytosolic chaperonin that folds approximately 10% of newly synthesized proteins and refolds denatured proteins under stress [18]. Each CCT has a structure of ~60 kDa and is predominantly involved in processing of cytoskeletal proteins [19]. Aberrant CCT expression has been shown to be associated with tumor progression. For instance, CCT3 can bind to Stat3 to accelerate its activation [20]. CCT8 could prevent the cell cycle arrest in colorectal cancer [21]. CCT2 was essential for antagonizing Gli-1 ubiquitination in colorectal cancer, especially under the hypoxic condition [22]. CCT8 and CCT6A could effectively facilitate THOC3 folding, and their knockdown reduced THOC3 expression. These findings suggest that THOC3 is a substrate protein encapsulated and folded by TRiC. Interfering with the interaction of TRiC might decrease the expression of THOC3.

Furthermore, THOC3 could bind to YBX1 in the cytoplasm, which is a classical m5C reader and responsible for RNA stability. We found that knockdown of THOC3 or YBX1 led to a significant decrease in the expression of 104 genes, including characters related to glycolysis or glucose metabolism [23], with PFKFB4 being markedly decreased. PFKFB4 is involved in glycolysis regulation and pentose phosphate production to exert carcinogenic function [24]. Loss of PFKFB4 induced cell cycle arrest and inhibited glycolysis [25]. PFKFB4 could accelerate the production of lactate and ATP to maintain the survival of cancer stem-like cells [26]. In addition, PFKFB4 could phosphorylate SRC to promote the progression of breast cancer and LUAD [27, 28]. Our study revealed that THOC3 regulated the nuclear-cytoplasmic shuttling of PFKFB4 mRNA, with binding sites mainly enriched in its 3′UTR. Once in the cytoplasm, THOC3 could form a complex with YBX1 to indirectly stabilize oncogenic transcripts. RNA pull-down assays showed that THOC3 knockdown attenuated the binding between YBX1 and PFKFB4 mRNA, resulting in the shorter half-life. PFKFB4 overexpression rescued THOC3-knockdown-inhibited LUSC progression. These finding propose that THOC3 inhibitors could suppress LUSC by decreasing PFKFB4 expression, and the addition of YBX1 inhibitors might provide better efficacy.

Moreover, YBX1 belongs to the RNA binding protein family with a cold-shock domain [14]. It is acknowledged that m5C participates in different stages of mRNA metabolism, including splicing, maturation, export, stability, translation, and decay [29]. By recognizing m5C, YBX1 could stabilize mRNA to promote tumor progression [15]. YBX1 also mediates the methylation that promotes the development of lung cancer [30, 31]. The current study confirmed the binding of YBX1 to 3’UTR of PFKFB4 mRNA and showed that YBX1 knockdown decreased the stability of transcription. The role of m5C in YBX1 binding was assessed using RNA pull-down assays with probes with or without m5C modification. The association between YBX1 and 3′UTR was impaired in the absence of m5C. Besides, m5C sites in PFKFB4 transcription were enriched in CG regions, consistent with the previous reports [32, 33]. NSUN2, a representative methyltransferase catalyzing m5C modification [34], was shown to facilitate the binding between YBX1 and PFKFB4 mRNA by RIP. Therefore, the m5C site in the 3′UTR of PFKFB4 mRNA was meaningful for mRNA stability, and YBX1 could specifically recognize this site to exert its function. Given the nature of YBX1, reducing the m5C modification may have the potential to inhibit tumor progression by blocking YBX1 binding.

## Conclusion

In summary, this study demonstrates that THOC3 promotes the proliferation, migration, and glycolysis of LUSC. The chaperonin TRiC, including CCT8 and CCT6A, modulates the folding of THOC3. THOC3 facilitates the nuclear export of PFKFB4 mRNA and cooperates with YBX1 to maintain its stabilization in the cytoplasm. YBX1 binds to the 3′UTR of PFKFB4 mRNA by recognizing m5C sites. THOC3 and YBX1 synergistically upregulated PFKFB4 to promote LUSC progression. Therefore, directly targeting THOC3 or disrupting its interaction with proteins or mRNA may have therapeutic potential for LUSC.

## Materials and methods

### Clinical specimens

We obtained the LUSC tissue microarray (TMA) (HLugS180Su02) from Shanghai Outdo Biotech and the final follow-up time was June 2016. A total of 90 LUSC tissue specimens and 90 normal tissue specimens were collected to compose the TMA. IHC was performed using an anti-THOC3 antibody (Santa Cruz, USA, sc-377456) and analyzed with automated quantitative analysis. The experimental protocol was approved by the Institutional Review Board of the First Affiliated Hospital of Nanjing Medical University (2023-SRFA-076). We confirmed that all study subjects provided signed informed consent, including detailed information about the purpose of the study, the procedures, the risks and potential benefits, and their right to withdraw consent and participation at any time.

### Bioinformatic analysis

We downloaded the gene expression data of LUSC cell lines from DEPMAP (https://depmap.org/portal/depmap/) [35]. The gene expression profile across all tumor samples and paired normal tissues was obtained from GEPIA (http://gepia.cancer-pku.cn/index.html) [36]. The RNA sequencing data and curated survival data of LUSC was downloaded from the database of TCGA (https://cancergenome.nih.gov/), GSE157011(*n* = 484) [37], and GSE73403 (*n* = 69). Binding sites between RNA binding proteins and RNA were predicted by catRAPID (http://service.tartaglialab.com/page/catrapid_group) [38].

### Cell lines

The human LUSC cell lines SK-MES-1, H1703, and H520, as well as the human normal pulmonary epithelial cell line B2B, were obtained from China Center Type Culture Collection (CCTCC, Shanghai). Authentication by STR profiling and confirmation of mycoplasma contamination-free status were performed prior to the experiments. Cells were cultured in RPMI 1640 (GIBCO, USA) medium supplemented with 10% fetal bovine serum (FBS), 100 U/ml penicillin, and 100 mg/ml streptomycin (Invitrogen, USA) at 37 °C in a 5% CO_2_ atmosphere. 5MPN was purchased from Beyotime and utilized at a concentration of 10 μM to inhibit the expression of PFKFB4.

### Cell proliferation

Cells were seeded in 96-well plates at a density of 2000 cells per well. Optical density (OD) values were measured at 450 nm using a CCK-8 assay (Beyotime, China) every 24 h for 4 days. For the colony formation assay, cells were seeded in 6-well plates at a density of 500 cells per well. After 1–2 weeks, cells were washed with PBS, fixed with 4% paraformaldehyde, stained with 0.1% crystal violet for 30 min, and washed twice. Colonies were photographed and counted under a light microscope.

### Cell migration assays

Cells were seeded at a density of 4 × 10^5^ cells/well in six-well plates for the wound healing assay. Scratches were made using a 10 µl pipette tip after adherence. Bright-field images of randomly selected views were captured twice every 12 h at 10× magnification. The cell migration rate was calculated using the formula: (mean remained breadth/mean wounded breadth) × 100%. Additionally, a transwell chamber was used for migration assay. Briefly, 5 × 10^4^ cells in 200 µl of serum-free medium were added to the upper chamber of an insert with an 8 µm pore size (Millipore). Medium with 20% FBS was added to the lower chamber. After 24 h incubation, migrating cells were fixed with 95% ethanol, stained with 0.1% violet crystal for 15 min, and counted under an inverted phase contrast microscope at a magnification of ×10. Five randomly selected fields were counted for each sample.

### Cell apoptosis assays

Flow cytometry was performed to measure cell apoptosis using FACS ARIA II SORP (BD Biosciences). 1 × 10^5^ cells were collected and labeled with the Annexin V-FITC/PI apoptosis detection kit for 30 min, following the manufacturer’s instructions (Beyotime, China). The samples were then analyzed by fluorescence activated cell sorter (FACS). Annexin V(+)/PI(−) denotes early apoptosis, while Annexin V(+)/PI(+) represents late apoptosis and necrosis.

### Glucose, lactic acid, and adenosine triphosphate (ATP) detection

After adherence, cells were incubated with fresh medium for 24 h (4 × 10^5^ cells/well). Intracellular levels of glucose and lactate were measured using a fluorescence-based glucose assay kit (Nanjing Jiancheng Bioengineering Institute, China) and a lactate test kit (KeyGEN BioTECH, China), respectively, following the manufacturer’s instructions. The total amount of glucose uptake and lactate acid production was normalized to cell number. ATP levels in cells (pmol/µg protein) were measured using an ATP colorimetric assay kit (Beyotime, China) according to the manufacturer’s instructions.

### RNA extraction and quantitative real-time PCR (qRT-PCR)

Total RNA was extracted from cell lines or tissues using an RNA extraction kit (Beyotime, China). Reverse transcription was performed using HiScript III RT SuperMix for qPCR (+gDNA wiper) (Vazyme, China) with 1 μg of total RNA. Amplification of synthesized cDNA was performed using HiScript III RT SuperMix for qPCR (Vazyme, China). β-actin was used as an internal control to normalize gene expression. The relative RNA levels of the indicated genes were calculated using the 2-ΔΔCT method. Each experiment was performed in triplicate. The primer sequences are listed in Supplementary Table S1.

### RNA stability assay

To investigate the stability of RNA, cells were treated with actinomycin D (2 µg/ml) and RNA was extracted at 1, 2, 4, and 6 h. RNA from untreated cells was also extracted at 0 h and served as the internal reference. The amount of RNA was quantified and plotted against time to determine the rate of RNA degradation.

### Cellular localization

The distribution of mRNA in cells was shown by Fluorescent In Situ Hybridization (FISH) Kit and probes (RiboBio, China). Pictures were taken by the confocal laser scanning microscope at a 40× magnification. Quantitative analysis was also performed by the cell nucleocytoplasmic separation kit (Beyotime, China) according to the manufacturer’s instructions. The distribution of proteins was determined by the IF assay using Immunol Fluorescence Staining Kit following the directions (Beyotime, China). Cells were fixed by 4% paraformaldehyde for 10 min and then permeabilized for 5 min by 0.5% Triton X-100. After 1 h blocking, cells were incubated with antibodies overnight and with fluorescent secondary antibodies for 2 h without light. DAPI was used for nuclear staining. Fixed cells were photographed to show subcellular locations.

### Transfection

siRNA and shRNA targeting specific genes were synthesized by Shanghai Genechem company (see Supplementary Table S1 for sequences). Full-length complementary DNAs (cDNAs) of human THOC3 and a negative control were also synthesized. Transfection was performed using Lipo8000™ Transfection Reagent (Beyotime, China) according to the manufacturer’s protocol. Lentivirus infection was screened with puromycin (1 μg/μl).

### RNA immunoprecipitation (RIP)

RIP was carried out using the EZ Magna RIP RNA-binding Protein Immunoprecipitation Kit (Millipore) with H1703 cell extracts. The following antibodies were used for immunoprecipitation: IP-grade anti-THOC3 (Santa Cruz, USA, sc-377456), anti-YBX1 (Proteintech, China, 20339-1-AP), and anti-Flag antibodies (CST, USA, #14793). Co-precipitated RNAs were quantified using qRT-PCR, and anti-IgG antibody was utilized as a negative control.

### RNA pull-down

The plasmids expressing PFKFB4 3′UTR and primers of the positive/negative-sense strand were designed by Zoonbio Co., Ltd. Biotin-labeled PFKFB4 3’UTR was transcribed in vitro using the Biotin RNA Labeling Mix and T7 RNA polymerase with the RNAmax-T7 biotin-labeled transcription Kit (RiboBio, China). The Pierce Magnetic RNA-Protein Pull-Down Kit (Thermo Fisher, USA) was used to extract RNA/protein complexes. Biotin-labeled RNA was co-incubated with cell lysates overnight, followed by incubation with the mixed RNA-protein binding reaction solution for 1 h. RNA/protein complexes were eluted and binding proteins were detected by Western blotting. Biotin-labelled RNA oligonucleotides with (oligo-m5C) or without m5C (oligo-C; 5′-biotin-TAGTXGGXGATAAGGTTGTGXGTGG-3′, X = C or m5C) were synthesized by Tsingke-Biotechnology Co., Ltd [15].

### m5C detection

RNA was extracted and converted using the EZ RNA Methylation Kit (Zymo Research, USA). Briefly, 1 μg of RNA was mixed with RNA Conversion Reagent and incubated at 70 °C for 5 min, followed by 54 °C for 45 min. The converted RNA was then desulphonated for 30 min. The resulting RNA was subjected to qRT-PCR amplification. Hot start enzyme was used for TA cloning amplification, and corresponding primers for transformation were designed (Supplementary Table S1). Sanger sequencing was performed by Shanghai Shenggong Biotechnology Co., Ltd. to detect the conversion of C to T.

### RNA-sequencing (RNA-seq)

RNA-seq was performed using the Illumina HiSeqTM2500 instrument by BGI Genomics (Beijing, China). Alignment and transcript splicing analyses were conducted using Tophat2 (http://ccb.jhu.edu/software/tophat/index.shtml) and Cufflinks software (http://cole-trapnell-lab.github.io/cufflinks), respectively. The fragments per kilobase million values were used for quantitative analysis of all genes, and differentially expressed genes were identified with *q* value < 0.005 and |log2(FoldChange)| > 1. The differentially expressed genes were subjected to Gene Set Enrichment Analysis (GSEA) and Kyoto Encyclopedia of Genes and Genomes pathway (KEGG) analysis. The glycolysis associated gene set was obtained from GSEA (M16864). Sufficient replicates were conducted to ensure statistical significance of the analysis. The sequencing data have been deposited to the SRA (PRJNA953801).

### Western blotting (WB)

The proteins were extracted using RIPA protein extraction reagent, and their concentrations were measured using the Detergent Compatible Bradford Protein Assay Kit (Beyotime, China). Subsequently, 10–15 µg of each protein sample was separated by 12% SDS-polyacrylamide gel electrophoresis (SDS-PAGE), transferred onto polyvinylidene fluoride membranes, and incubated with a blocking buffer. The membranes were then incubated with primary antibodies, followed by secondary antibodies, and protein levels were detected using an enhanced chemiluminescence reagent (xinsaimei, China). Antibodies were purchased from manufacturers: THOC3 (Santa Cruz, USA, sc-377456), YBX1 (Proteintech, China, 20339-1-AP), Flag (CST, USA, #14793), β-Actin (CST, USA, #3700), PFKFB4 (Bioss, China, bs-12632R), NSUN2(Bioss, China, bs-19477R), CCT8 (Santa Cruz, USA, sc-377261), CCT6A(Santa Cruz, USA, sc-514466), and CCT2 (Santa Cruz, USA, sc-374152).

### Co-immunoprecipitation assays (Co-IP)

Protein extracts were incubated overnight with specific primary antibodies and Protein A/G PLUS-Agarose beads (Santa Curz, USA). The beads were then washed with lysis buffer or PBS, and eluted with SDS-PAGE buffer. The eluted proteins were detected using WB. Flag-tagged proteins were precipitated using the Flag-tag Protein IP Assay Kit with Magnetic Beads according to the manufacturer’s instructions (Beyotime, China).

### Mass spectrometry analysis

Co-IP proteins were visualized by Fast Silver Stain Kit (Beyotime, China). The protein bands of interest were excised from the gel and subjected to in-gel trypsin digestion. Resulting peptides were extracted and analyzed by nanoLC-MS/MS (Sangon Biotech, Shanghai, China) using an Orbitrap Fusion mass spectrometer. The shotgun strategy was employed for proteomics studying. Protein identification was performed using Mascot search engine and the SwissProt database. The mass spectrometry proteomics data have been deposited to the ProteomeXchange Consortium (http://proteomecentral.proteomexchange.org) via the iProX partner repository [39, 40] with the dataset identifier PXD041447.

### Xenograft model in nude mice

Animal experiments with a sample size of 5 per group was determined based on the probability level of 0.05 and power of 0.80. On the basis of previous study [41], an expected 80% difference in the effect of gene with a coefficient of variation of 40% yielded a minimum sample size of five mice for each group. About 1 × 10^6^ cells were dissolved in 100 µl PBS and injected subcutaneously into the axilla of BALB/c nude male mice (4 weeks old). After the numbering, the generated random sequence was matched to the animal number, and the animals were grouped according to the random sequence. If the mice develop serious health problems or tumor over 2 cm^3^, they are excluded from the analysis. Tumor volumes were calculated by measuring the longitudinal and latitudinal diameters every week. After 3 or 4 weeks, the mice were euthanized, and their tumors were isolated and fixed in 4% formalin for IHC analysis. Lung burden was detected one month after the tail vein injection with tumor cell suspension (1 × 10^6^ cells/100 μl). Animal experiments were not conducted in a double-blind manner. The protocol was approved by the Committee on the Ethics of Animal Experiments of the Nanjing Medical University (IACUC-2201042).

### Immunohistochemical staining

IHC analysis was performed to evaluate the expression of Ki-67 (Servicebio, China, GB11030), THOC3 (Santa Cruz, USA, sc-377456), and PFKFB4 (Bioss, China, bs-12632R) by corresponding antibodies at a dilution of 1:100. The staining was visualized with DAB (3,3′-diaminobenzidine) substrate kit (Beyotime, China) according to the manufacturer’s instructions. Slides were examined independently using ImageJ software.

### Statistics

All in vitro experiments were performed in triplicate and repeated three times. The sample size was determined based on a power analysis, considering the desired power level and alpha level. Statistical significance in cell proliferation, migration, and invasion assays was assessed using a paired Student’s *t* test, *χ*2 test, Wilcoxon test, or one-way analysis of variance (ANOVA), as appropriate. Homogeneity of variance between groups was statistically compared. Statistical analyses were performed using SPSS version 20.0 software or GraphPad Prism 8.0 software. Difference and correlation analyses were conducted using R software (version 4.1.1, www.r-project.org). *P* values less than 0.05 were considered statistically significant. The results presented are representative of one experiment out of three independent trials, and the data are expressed as means ± standard deviation. *P < 0.05; **P < 0.01; ***P < 0.001.

## Supplementary information

supplementary material

Original Data File

checklist

supplementary table S1

supplementary table S2

supplementary table S3

## Data Availability

The R code used to analyze the data in this paper is available upon reasonable request. Interested investigators may contact the corresponding author for access to the code.

## Data availability

Data are available on reasonable request.
