# Supplementary material for: THOC3 interacts with YBX1 to promote lung squamous cell carcinoma progression through PFKFB4 mRNA modification
Source: Cell Death Dis. 2023 Jul 27;14(7):475. doi: 10.1038/s41419-023-06008-3 (PMC10374565; doi:10.1038/s41419-023-06008-3)
Supplement: Supplementary file 1 — supplementary material [file 41419_2023_6008_MOESM1_ESM.docx]

Supplementary Figures


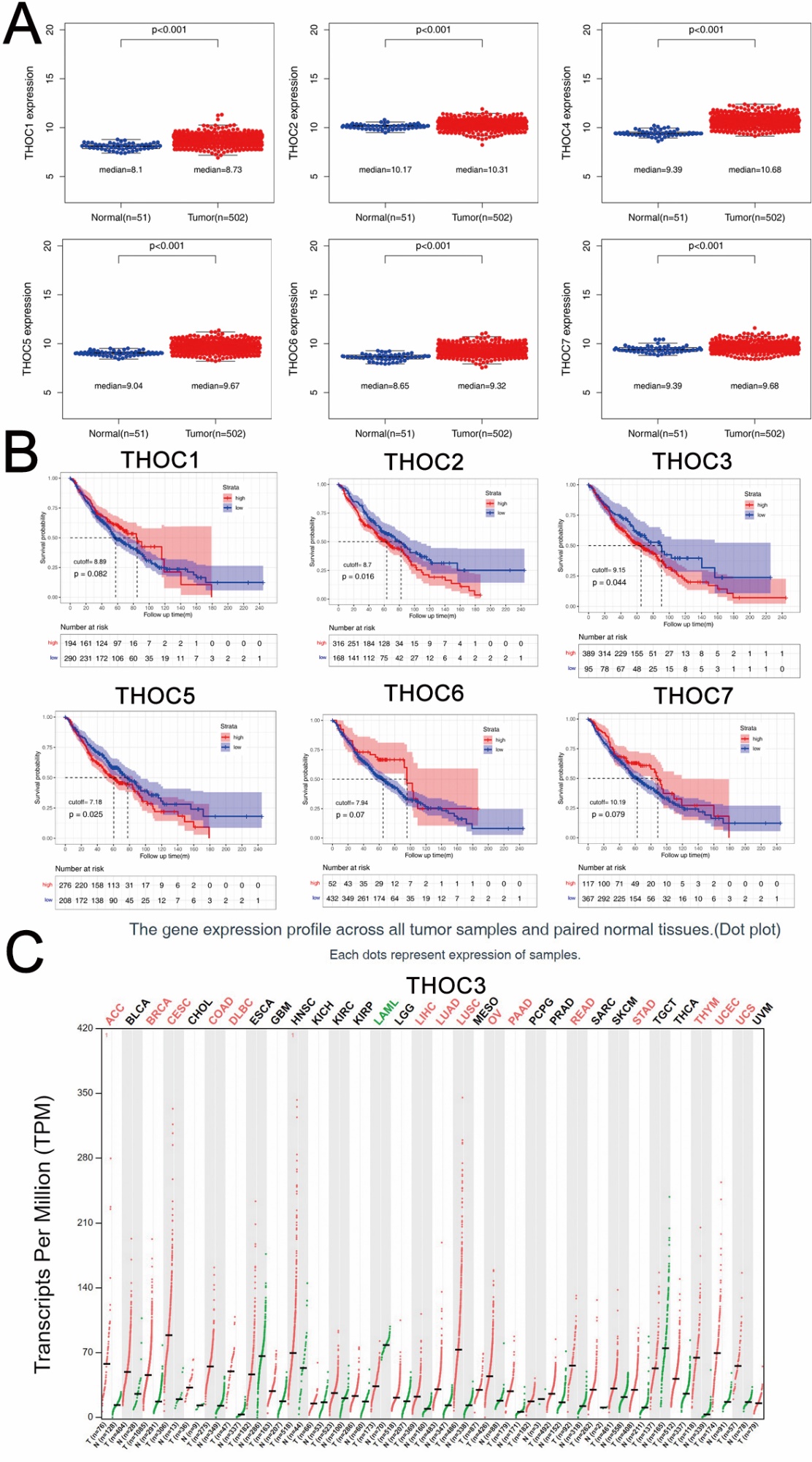


**SFig.1** **A.** Expression of THOC1/2/4/5/6/7 in LUSC (n=502) and normal tissues (n=51) on the basis of TCGA. **B.** Kaplan-Meier plot of patients recruited in GSE157011 with high (red) or low (blue) expression of THOC1/2/3/5/6/7 (*P* =0.082, 0.016, 0.044, 0.025, 0.07, and 0.079, respectively). Cutoff values were automatically determined. **C.**THOC3 expression in 31 types of tumors and adjacent normal tissues obtained from GEPIA.


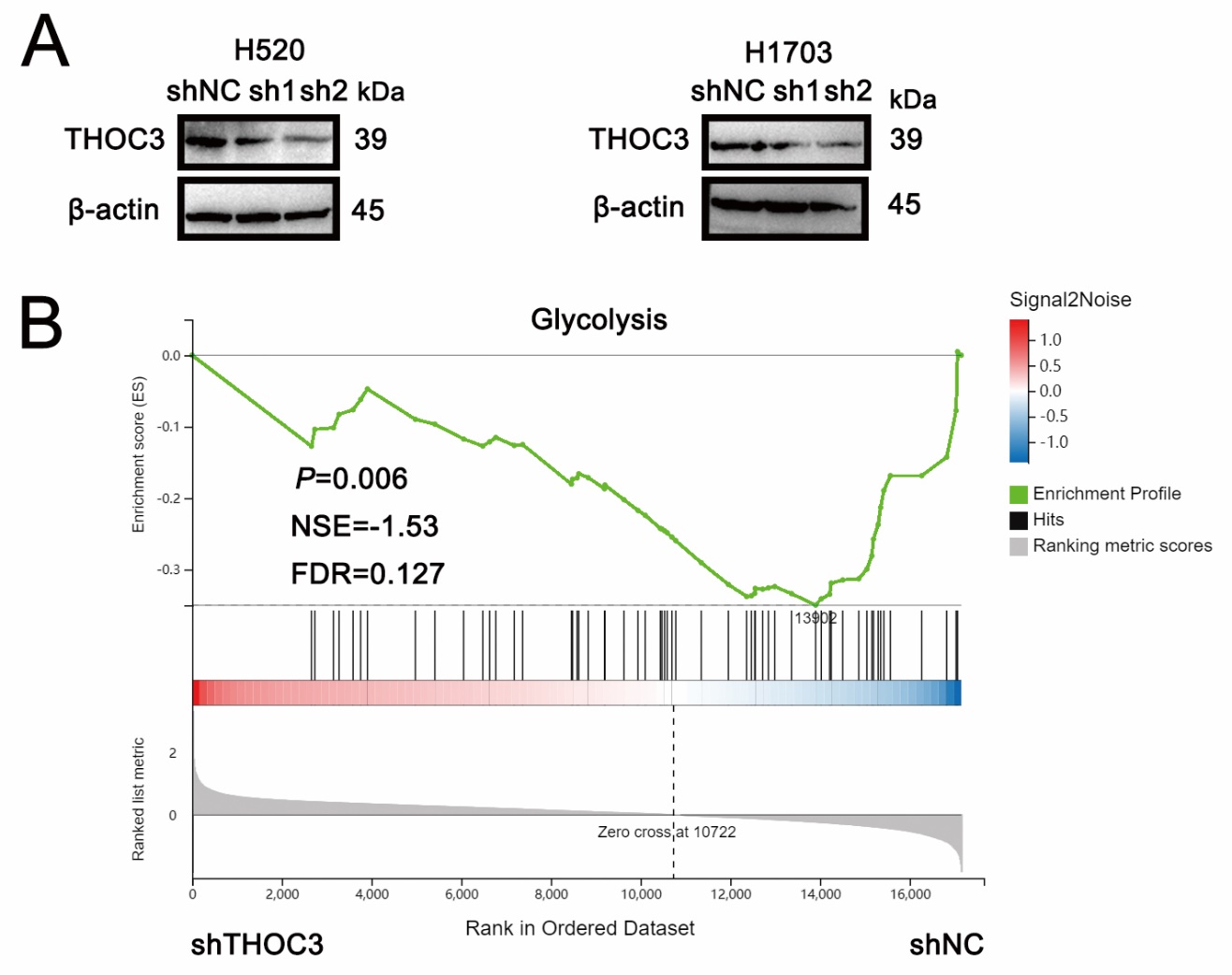


**SFig.2** **A.** Expression of THOC3 in H520/H1703 infected with sh-NC and THOC3-sh1/2 shown using qRT-PCR and WB. **B.** GSEA revealed that the glycolysis pathway was significantly enriched in the negative control group compared with the THOC3 knockdown group (*P* =0.006).


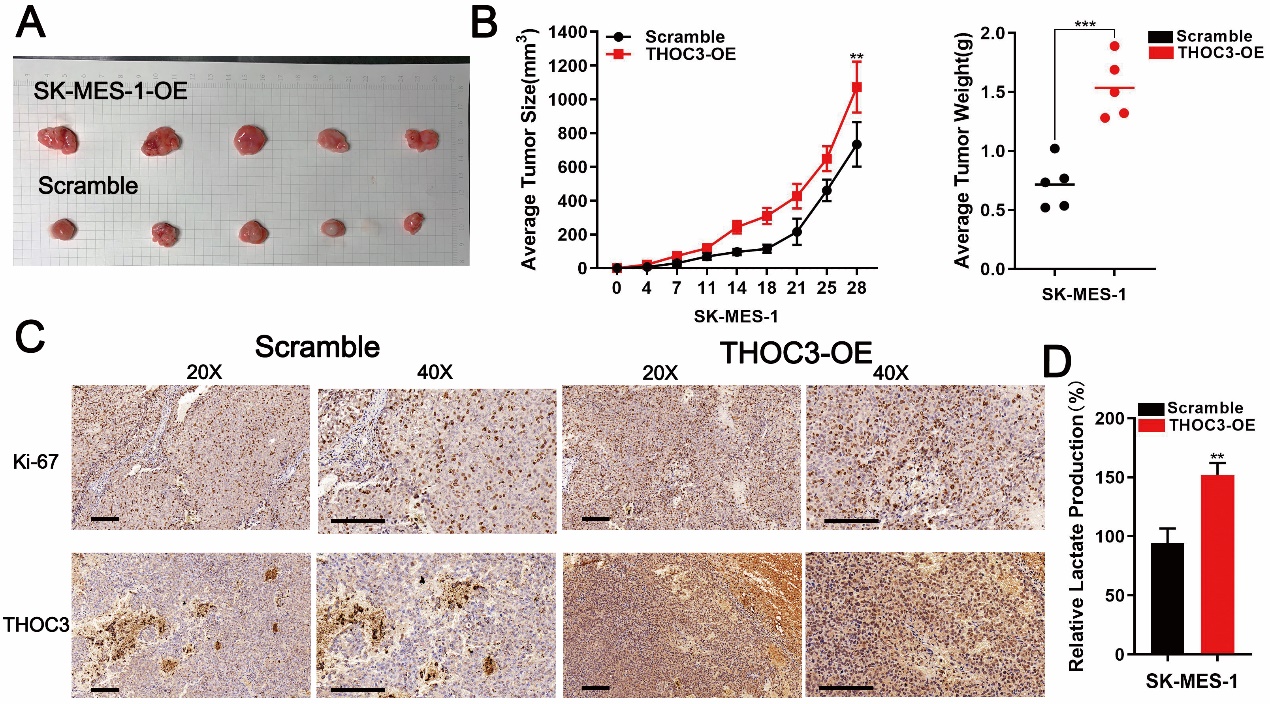


**SFig.3 A.** Representative images of subcutaneous tumors from mice injected with 1 × 10^6^ THOC3 overexpressed/Scramble SK-MES-1 cells on the left flank (n=5, each group). **B.** The mean weight of each tumor is measured (left); tumor growth curves are drawn per week. **C.** Levels of Ki-67 index and THOC3 in Scramble/THOC3-OE groups were shown by IHC (20× and 40× magnification). Scale bars = 100 μm. **D.** Lactic acid production of the THOC3-OE group compared with the Scramble group. **P* < 0.05; ***P* < 0.01; ****P* < 0.001. Variables are presented as mean ± SD.


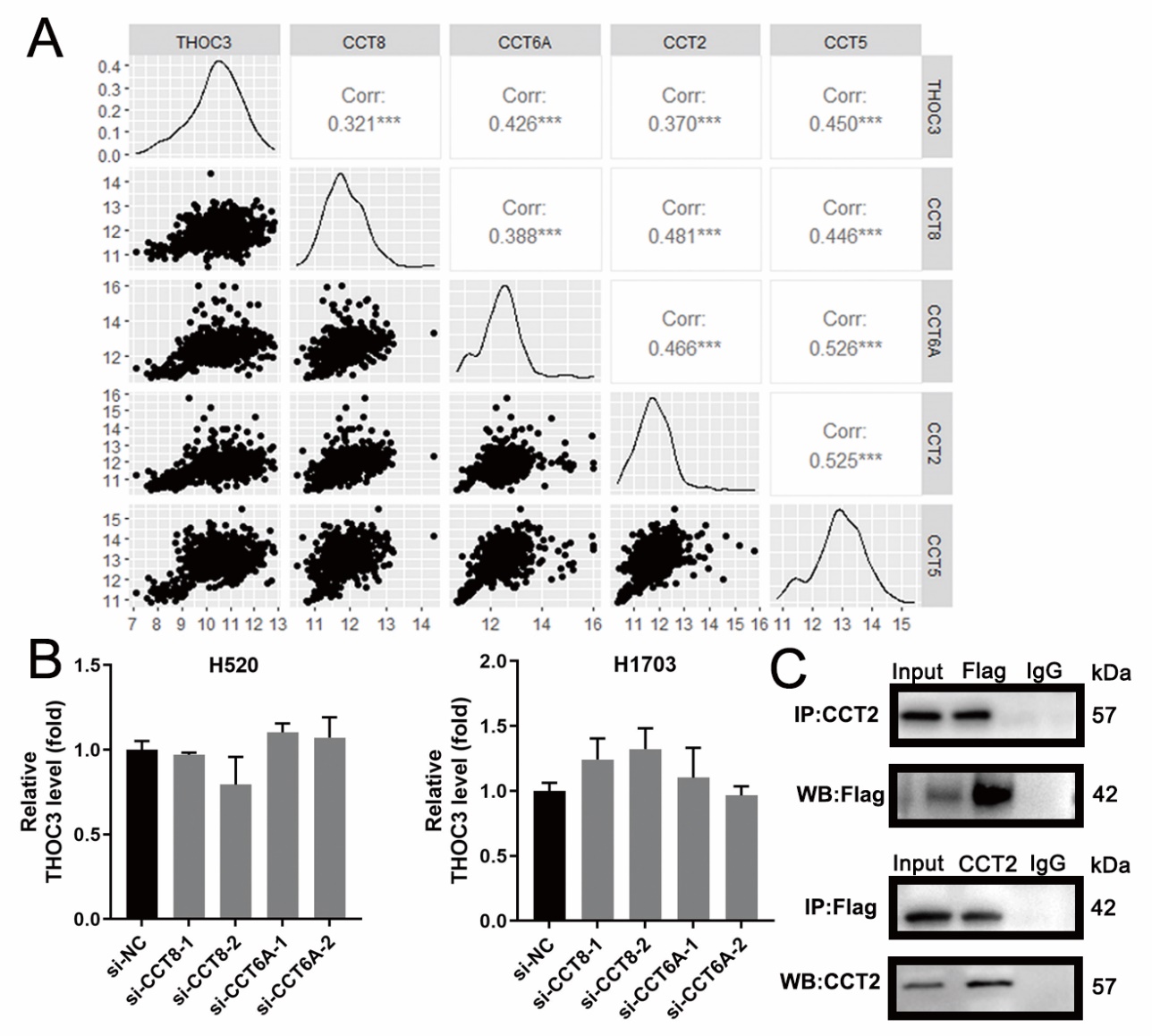


**SFig.4** **A.** Correlation analysis of THOC3 with CCT8, CCT6A, CCT2, and CCT5 in TCGA cohort. **B.** THOC3 mRNA expression in cells transfected with si-NC or si-CCT8/CCT6 shown via qRT-PCR. **C.** Cell lysates of H1703 transfected with Flag-tagged THOC3 underwent immunoprecipitation using anti-CCT2, anti-Flag, or IgG antibody, followed by immunoblotting with anti-Flag/CCT2 antibodies. **P* < 0.05; ***P* < 0.01; ****P* < 0.001. Variables are presented as mean ± SD.


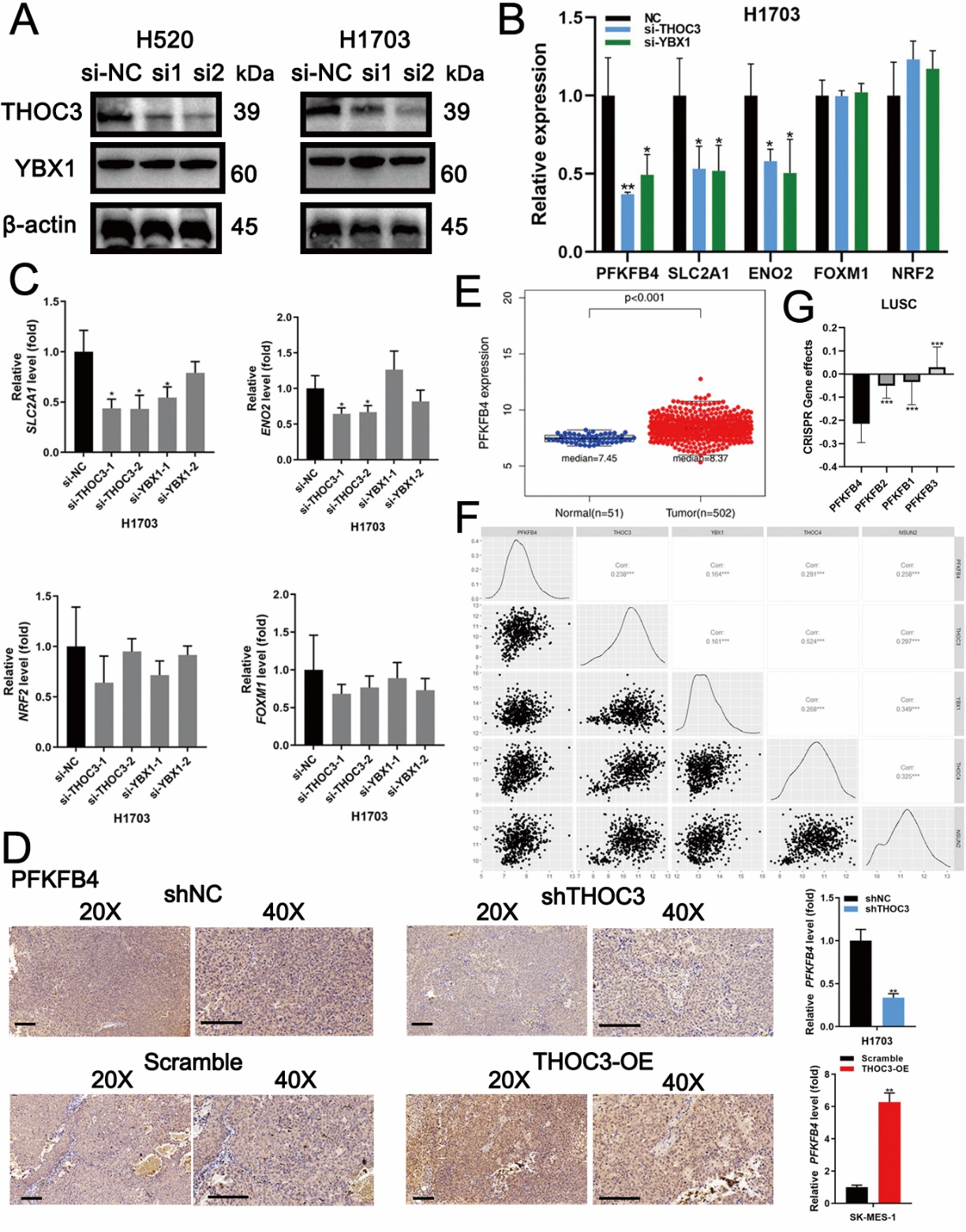


**SFig.5 A.** THOC3 and YBX1 expression in cells transfected with si-NC or THOC3 si1/2 shown by WB. **B.** Expression of PFKFB4, SLC2A1, ENO2, FOXM1, and NRF2 mRNA in H1703 transfected with si-NC or si-THOC3/YBX1 shown by RNA-seq. **C.** Expression of SLC2A1, ENO2, NRF2, and FOXM1 mRNA in cells transfected with si-NC or si-THOC3/YBX1 shown by qRT-PCR. **D.** Levels of PFKFB4 in shNC/shTHOC3 and Scramble/THOC3-OE groups shown by IHC (20× and 40× magnification). Scale bars = 100 μm (left); PFKFB4 mRNA in shNC/shTHOC3 H1703 and Scramble/THOC3-OE SK-MES-1 cells shown by qRT-PCR (right). **E.** Expression of PFKFB4 in LUSC (n=502) and normal tissues (n=51) on the basis of TCGA. **F.** Correlation among PFKFB4, THOC3, THOC4, YBX1, and NSUN2 in TCGA cohort. **G.** Effects of PFKFB1/2/3/4 knockout by CRISPR/Cas9 on 22 LUSC cell lines according to DEPMAP. **P* < 0.05; ***P* < 0.01; ****P* < 0.001. Variables are presented as mean ± SD.


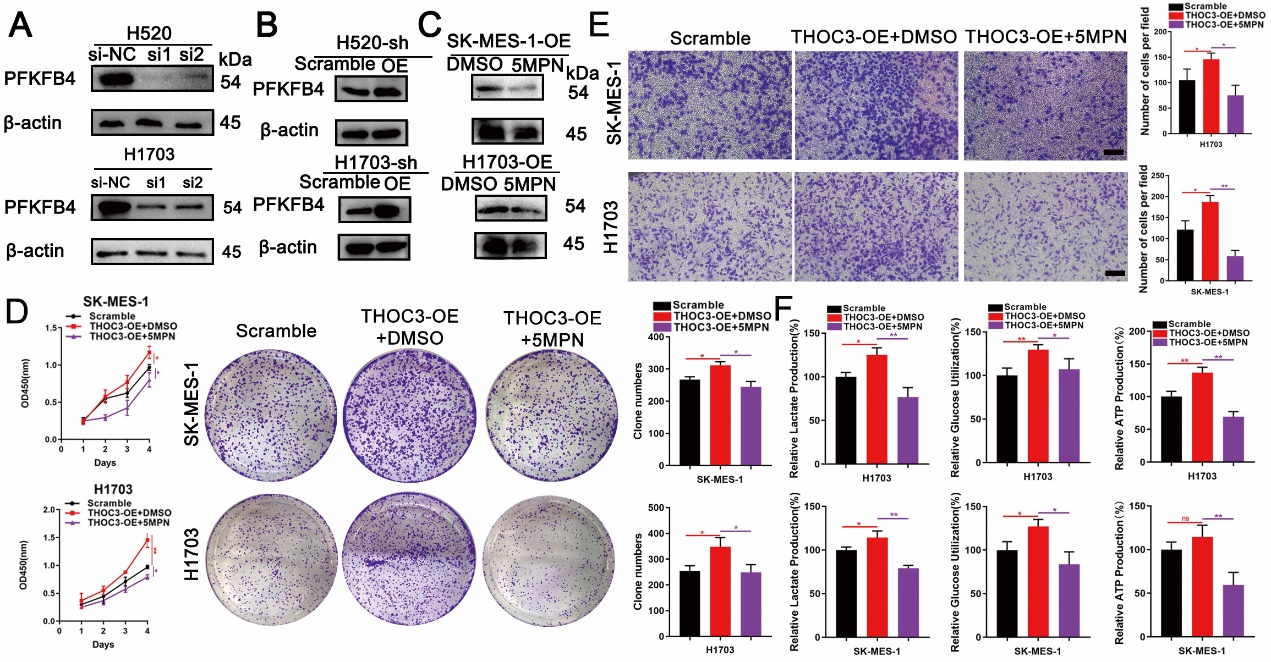


**SFig.6 A.** WB showing the expression of PFKFB4 in H520/H1703 cells transfected with si-NC or PFKFB4 siRNAs. **B.** WB showing the expression of PFKFB4 in THOC3 knockdown H520/H1703 cells transfected with scramble or PFKFB4-OE plasmids. **C.** WB showing the expression of PFKFB4 in THOC3 overexpressed SK-MES-1/H1703 cells with DMSO or 5MPN (10μM). **D.** Growth curves (days 1-4) and colony formation assays show the proliferation of THOC3-OE SK-MES-1/H1703 cells with DMSO or 5MPN (10μM). **E.** Transwell assays were conducted to assess cell migration of THOC3-OE SK-MES-1/H1703 cells with DMSO or 5MPN (10μM), with cells crossing the membrane stained with crystal violet (10× magnification). Scale bars = 100 μm. **F.** The relative glucose uptake, lactic acid production, and ATP concentration of THOC3-OE SK-MES-1/H1703 cells with DMSO or 5MPN (10μM) after 24 h. **P* < 0.05; ***P* < 0.01; ****P* < 0.001. Variables are presented as mean ± SD.


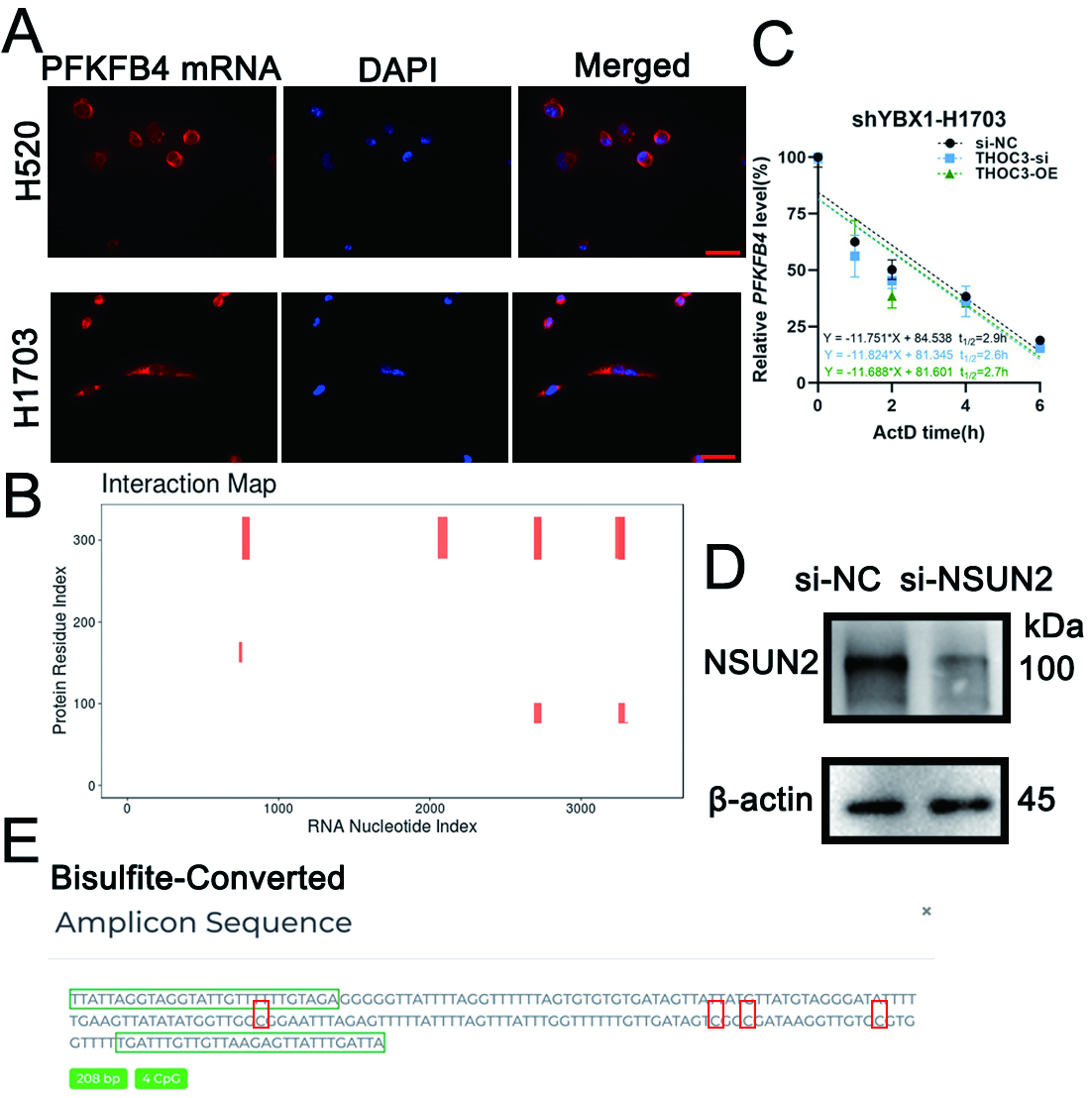


**SFig.7 A.** Representative images of PFKFB4 mRNA localization in H520/H1703 cells detected using FISH (40× magnification). **B.** THOC3 binding sites in PFKFB4 mRNA predicted by catRAPID. **C.** After actinomycin D treatment, PFKFB4 mRNA is detected at indicated time points in shYBX1-H1703 transfected with si-NC, THOC3-siRNA, or THOC3-overexpressed plasmids. **D.** WB showing NSUN2 expression in cells transfected with si-NC or si-NSUN2. **E.** Targeted amplicon sequence of PFKFB4 3’UTR predicted by ZYMO research.


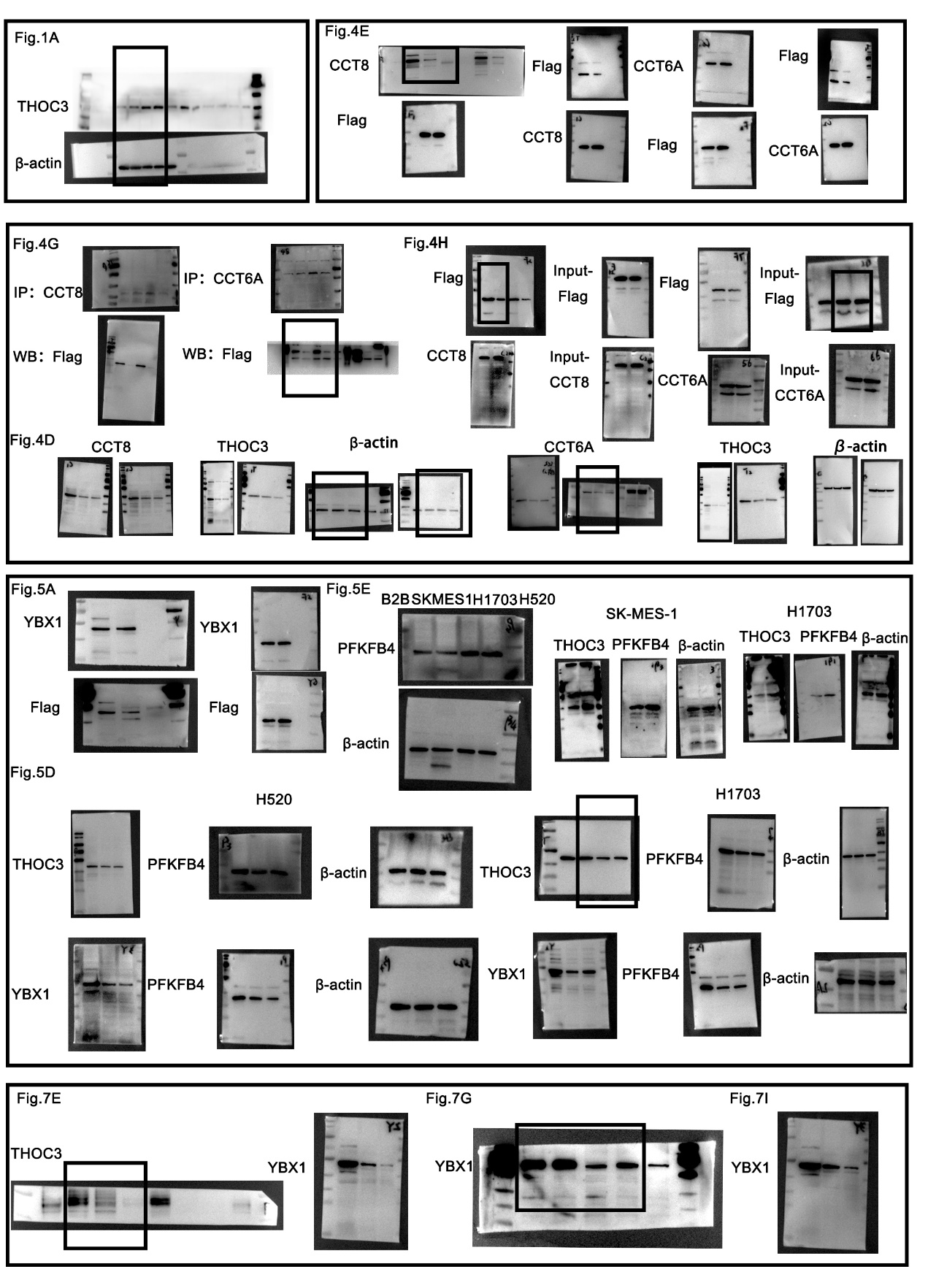

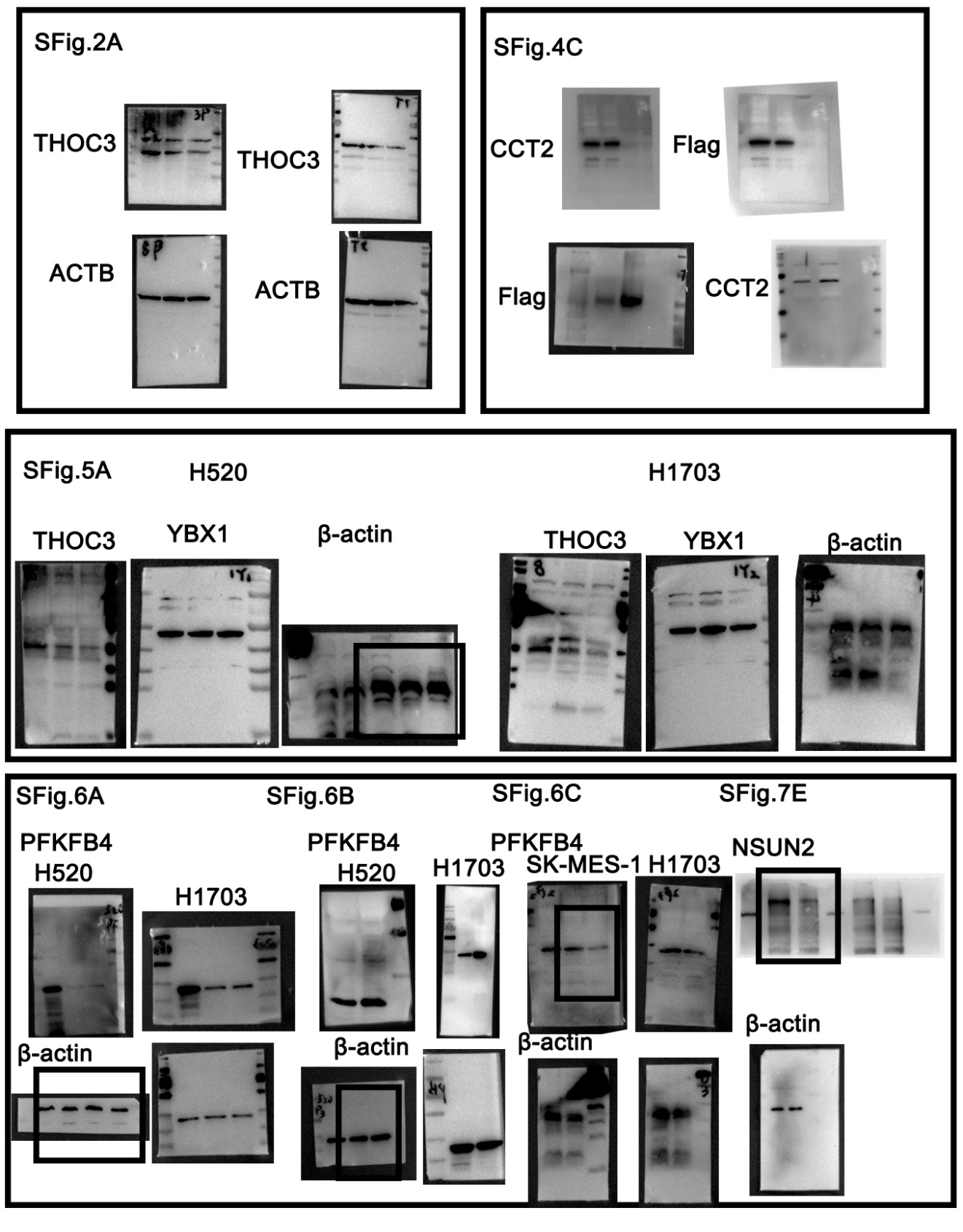


**Figure S8.** Unprocessed scans of western blot analysis. Some immunoblotting assays membranes are cut into pieces to incubate with different antibodies. Hence, the raw images of these membranes are of small size.

**Supplementary** **Tables**

**Table S1.** Primers used for qPCR, amplification, and siRNAs or shRNA oligonucleotides.

**Table S2.** Proteins immunoprecipitated by THOC3 with unused scores over 5 shown by mass spectrometry analysis and following GO analysis.

**Table S3.** Genes significantly downregulated upon THOC3 or YBX1 knockdown.

Sheet1: Genes significantly downregulated upon THOC3 knockdown (n=169).

Sheet2: Genes significantly downregulated upon YBX1 knockdown (n=411).

Sheet3: Genes associated with the carbohydrates metabolism from GSEA (M16864) (n=293).
