# Supplementary material for: THOC3 interacts with YBX1 to promote lung squamous cell carcinoma progression through PFKFB4 mRNA modification
Source: Cell Death Dis. 2023 Jul 27;14(7):475. doi: 10.1038/s41419-023-06008-3 (PMC10374565; doi:10.1038/s41419-023-06008-3)
Supplement: Supplementary file 2 — Original Data File [file 41419_2023_6008_MOESM2_ESM.docx]

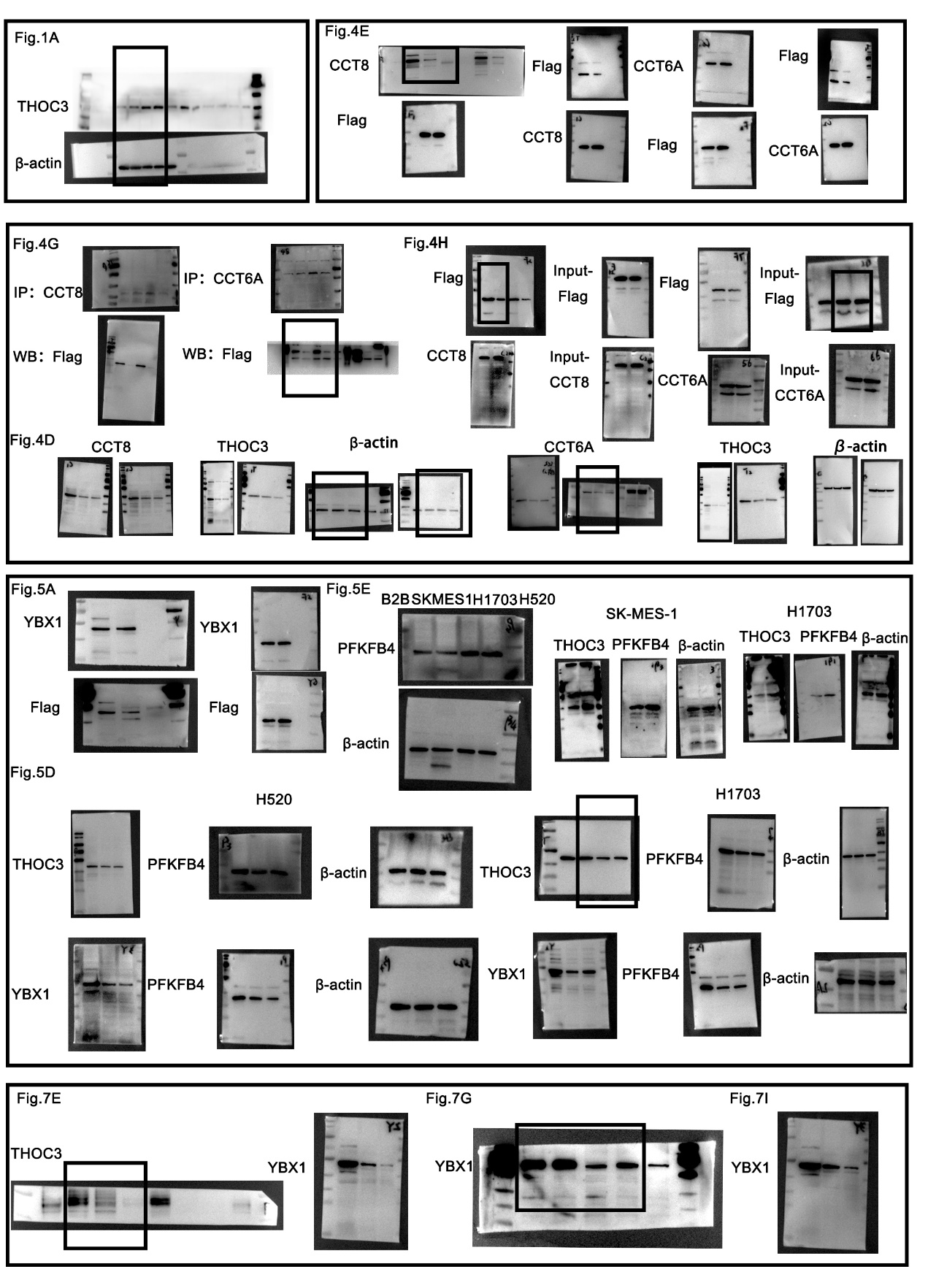


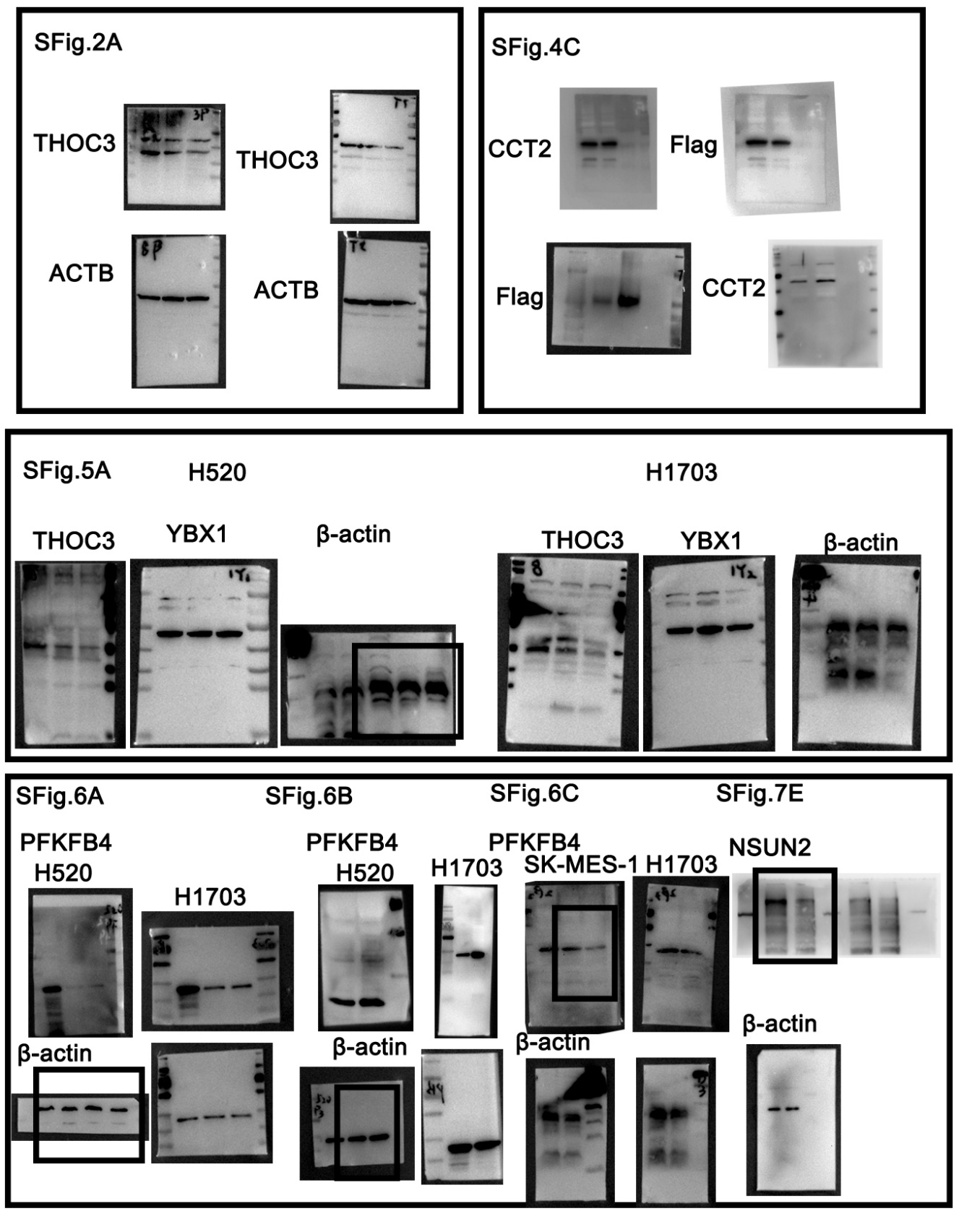


Unprocessed scans of western blot analysis. Some immunoblotting assays membranes are cut into pieces to incubate with different antibodies. Hence, the raw images of these membranes are of small size.
